# 3D time‐varying simulations of Ca^2+^ dynamics in arterial coupled cells: A massively parallel implementation

**DOI:** 10.1002/cnm.2786

**Published:** 2016-07-01

**Authors:** Constantine Zakkaroff, Stephen Moore, Stewart Dowding, Tim David

**Affiliations:** ^1^ UC HPC CentreUniversity of CanterburyChristchurchNew Zealand; ^2^ IBM Research Collaboratory for Life SciencesMelbourneAustralia

**Keywords:** coupled arterial cells, endothelial cells, smooth muscle cells, intercellular calcium waves, atherosclerosis, large‐scale physiological simulation

## Abstract

Preferential locations of atherosclerotic plaque are strongly associated with the areas of low wall shear stress and disturbed haemodynamic characteristics such as flow detachment, flow recirculation and oscillatory flow. The areas of low wall shear stress are also associated with the reduced production of adenosine triphosphate in the endothelial layer, as well as the resulting reduced production of inositol trisphosphate (IP^3^). The subsequent variation in Ca^2+^ signalling and nitric oxide synthesis could lead to the impairment of the atheroprotective function played by nitric oxide. In previous studies, it has been suggested that the reduced IP^3^ and Ca^2+^ signalling can explain the correlation of atherosclerosis with induced low WSS and disturbed flow characteristics. The massively parallel implementation described in this article provides insight into the dynamics of coupled smooth muscle cells and endothelial cells mapped onto the surface of an idealised arterial bifurcation. We show that variations in coupling parameters, which model normal and pathological conditions, provide vastly different smooth muscle cell Ca^2+^ dynamics and wave propagation profiles. The extensibility of the coupled cells model and scalability of the implementation provide a solid framework for *in silico* investigations of the interaction between complex cellular chemistry and the macro‐scale processes determined by fluid dynamics. © 2016 The Authors. International Journal for Numerical Methods in Biomedical Engineering published by John Wiley & Sons Ltd.

## 1 Introduction

The recently coined term *in silico* research refers to computer simulations of complex biological systems dynamics. This research approach offers the potential of increasing the speed of knowledge discovery 1, 2. *In silico* experiments have the potential to provide insight into the observations obtained by the experimental science. In the context of biological systems dynamics, the *in silico* simulations enable the rapid pruning of the parameter search space for the refinement and integration of cellular‐level models into biologically realistic macro‐scale models. The large‐scale physiological simulations described here were designed to provide insight into the effects of the luminal concentration variations on adenosine triphosphate (ATP)‐dependent dynamics in the coupled endothelial cells (ECs) and smooth muscle cells (SMCs) making up an arterial wall. The simulations of this nature provide a unique opportunity to perform experiments, which would never be possible in the *in vivo* and *in vitro* settings. For example, various specific pathological conditions, as described further in the text, can be simulated by changing the homocellular and heterocellular coupling parameters. Numerical simulations of this nature have never been attempted before at the scale of millions of coupled cells.

The arterial wall consists of three layers of tissue: *tunica intima*, *tunica media* and *tunica adventita*. The *intima* consists of a single layer of ECs; the *media* consists of the SMCs, and the *adventita* is made up of connective tissue. The endothelial layer plays the role of a mediator in the transfer of chemical species to and from the surrounding tissue. The mass transport phenomenon where chemical species are actively or passively diffused within large populations of cells is made possible through cellular communication channels. These channels are known as gap junctions; they are composed of connexin protein subunits and allow direct intercellular communication. For example, the Cx37 homotypic connexins are highly selective and allow only the transfer of monovalent cationic currents; thus, they have the most influence on the membrane potential 3. The Cx40 connexin protein subunit favours divalent cations, and thus, it is the major contributor to a gradient‐driven Ca^2+^ concentration current. The Cx43 connexin is the least selective, and it allows the passage of a range of large and small molecules such as ATP and inositol triphosphate (IP^3^) 4. The Cx43 connexin is normally present only in ECs although it has been reported to appear at the shoulder regions of the atherosclerotic lesions 5.

Recent publications on the physiology of vascular system dynamics hypothesise that cellular Ca^2+^ concentration oscillations (spreading both downstream and upstream) triggered by low WSS play an important role in atherosclerotic plaque formation 6, 7, 8, 9. The outcomes of the published *in vivo* and *in vitro* experiments provide descriptive models that use cellular‐level observations, but these observations fall short of explaining the macro‐scale phenomena of plaque formation and development 10. Atherosclerotic plaque lesions have characteristic‐length scales much larger than a single cell in the arterial wall. Hence, the mechanisms of plaque formation must be studied over considerable distances, such as hundreds to thousands of cell lengths.

### 1.1 Emergent macro‐scale behaviour

On the macro‐scale level, local geometric properties of arterial trees such as curvature, branching and bifurcations determine the local haemodynamics properties such as pressure, flow velocity and WSS. Among those properties, WSS carries a special significance as it influences the localisation of atherosclerotic lesions 11. Recent studies have established a link between atherosclerosis and low WSS, resulting from disturbed flow 12, 13. The magnitude of WSS determines the ATP concentration in the luminal boundary layer, which is in constant contact with the endothelium 14, 15. ATP is often described as the chemical energy transporter in cellular metabolism, and in the case of the vascular dynamics, it plays the role of a vasoconstrictor. In other studies, it has been proven that reduced ATP concentration is correlated with low WSS 16. Attempting to explain the onset of atherosclerosis, a study by 17 has proposed that atheroprotective mechanisms in a vascular wall are inhibited by reduced IP^3^ and Ca^2+^ signalling induced by low WSS.

The work of 9 presents a comprehensive example of cellular dynamics modelling, focusing on Ca^2+^ wave propagation in the SMC layer of arterial wall. This example of cellular dynamics modelling is aligned with the work presented here because 9 take into consideration the properties of WSS 8. However, the research presented in this article differs in two major ways from the approach of 9. Firstly, the model reported here is a coupled cells model, which includes both SMC and EC layers, and is therefore physiologically correct and able to interpret the transduction properties of the endothelial layer. Secondly, the macro‐scale phenomena of cell coupling are modelled on the micro‐scale where the ECs and SMCs in the arterial wall have physiologically accurate dimensions.

This study investigates temporal and spatial cellular dynamics of large populations of coupled ECs and SMCs with the view of gaining insight into the Ca^2+^ signalling in arterial wall tissue. The basic EC/SMC coupled model 18 has been validated through *in vivo* experiments in the work of 19. The experiments were performed with SMC arterial tissue strips of 750 × 50*μ*m, which were equivalent to populations of approximately 140 SMCs. The *in silico* experiments reported in the work of 20 were performed with the population of about 400 SMCs. Our simulations included the populations of 850 000 SMCs and 330 000 ECs, which total is approximately 1.2 millioncoupled cells. These simulations provide new evidence over much larger scales equivalent to that found for atherosclerotic plaques.

The presented work is an initial investigation into the relationship between micro‐scale cellular dynamics and the resulting ‘emergent behaviour’ that occurs on a macro‐scale exemplified by the size of atherosclerotic plaques. There is a clear correlation between the dynamics and the increase in intimal hyperplasia as found in *in vitro* and *in vivo* (cadaver) studies. Indeed, there is considerable evidence in the work of 12 and 21 demonstrating that eNOS and the production of NO is reduced in areas of low WSS, which in turn leads to endothelial dysfunction 22, resulting in the increased probability of atherosclerotic plaque formation. The eNOS‐mediated production is a function of cytosolic calcium, which is available from the stores through the IP^3^‐mediated channel. IP^3^ concentration is determined by the P2Y receptor mediated by agonists such as ATP. Finally, we note that as early as 1988 23, there was found a clear relationship between WSS/calcium‐induced potassium currents; these biochemical changes were hypothesised to be related to atherosclerotic regions 11, 24. In our work, we explore the relationship of calcium dynamics and arterial geometry as a way of determining how spatially and time‐varying agonist concentration can produce calcium transients over much larger scales than a single cell, giving credence to the idea that plaques grow both upstream and downstream. This work offers the possibility of understanding further the process of the initiation and growth of atherosclerotic plaques.

### 1.2 Resistance arterioles versus coronary artery simulations

The majority of experiments showing electrical signalling are conducted using small diameter vessels either denuded of endothelium or vessels that contain only ECs 25, pressurised vessels without fluid flow 26 or that the current through the gap junction remains constant 25. These experiments also do not show oscillatory behaviour as a function of space because this is difficult if not impossible to replicate under laboratory conditions. These constraints do not occur in our simulations, thus allowing for a more physiologically correct simulation.

The bi‐directional relationship between Ca^2+^ and WSS is a lot more prominent in small diameter resistance arteries. Vasomotion is not considered in this work because this phenomenon occurs more readily in resistance vessels rather than, say, the coronary tree. The focus of the paper is on much larger atherosclerosis prone vessels such as the coronary where contractility does not vary as much as that seen in smaller resistance arterioles. Fluid dynamics calculations show that WSS varies little in terms of the dilation or contraction of the artery (unless it is a small resistance vessel) compared with the base WSS induced by the vascular geometry of a bifurcation.

### 1.3 Parallel coupled cells simulations

The macro‐scale simulations reported in this work were implemented on the Blue Gene parallel architecture where a number of coupled EC/SMC units are grouped into a single task/domain in the parallel computational environment. These tasks interact with each other by exchanging state variable values for the neighbouring cells along the edge of the computational domain at every time step during the simulation process. The interaction between the groups of coupled EC/SMC units was implemented through the MPI. Full details of the computational solution are provided in Section 2.3.

In this article, Section 2 briefly reviews the model for a single pair of coupled ECs/SMCs, which is based on the work of 18. The mass transfer dynamics are modelled by systems of non‐linear ordinary differential equations (ODEs). The implementation of this coupled‐cell model along with initial simulations has been previously described in the work of 27. The core of the computation model described here is a single‐coupled EC/SMC unit modelled by a system of nine ODEs with four and five equations for an EC and SMC, respectively.

The simulations reported in this work are divided into two groups. The first group is based on a synthetic ATP agonist map generated with a sigmoid function parametrised with the distance to the origin of the vessel segment, while the second group is based on the CFD‐based agonist map generated from a model of physiologically‐realistic mass transfer simulation of blood flow in a vessel. The results for both simulation groups are provided in Section 3, followed by discussion and conclusions in Section 4 and Section 5, respectively.

## 2 Methods

The experimental methods described here extend the work reported previously by 27. An overview of the coupled interactions between SMCs and ECs is provided in Section 2.1. The coupled cells physiological model remains unchanged; a brief review of the model is provided in Appendix  A.1 for completeness. The cell coupling parameter variations in which were used in the simulations reported here are provided in Section 2.2. The computational solution, however, has been significantly extended to allow the modelling of arterial segments with complex geometric features such as branching and bifurcations. The critical difference from the computational solution presented by 27 is the 2D Cartesian communication between the groups of coupled cells comprising the discrete domains of the vessel surface. This Cartesian model of inter‐domain communication supports coupled cells simulations with physiologically realistic arterial segments; the enhanced arterial surface generation is based on a method for parametric modelling of bifurcating structures. The computational solution description is provided in Section 2.3, including the Cartesian surface modelling and macro‐scale inter‐cellular communication. Section 2.4 describes the generation of synthetic ATP agonist maps, while Section 2.5 describes the CFD‐based modelling of the ATP simulation inputs.

### 2.1 Overview of a coupled cells unit

**Figure 1:**
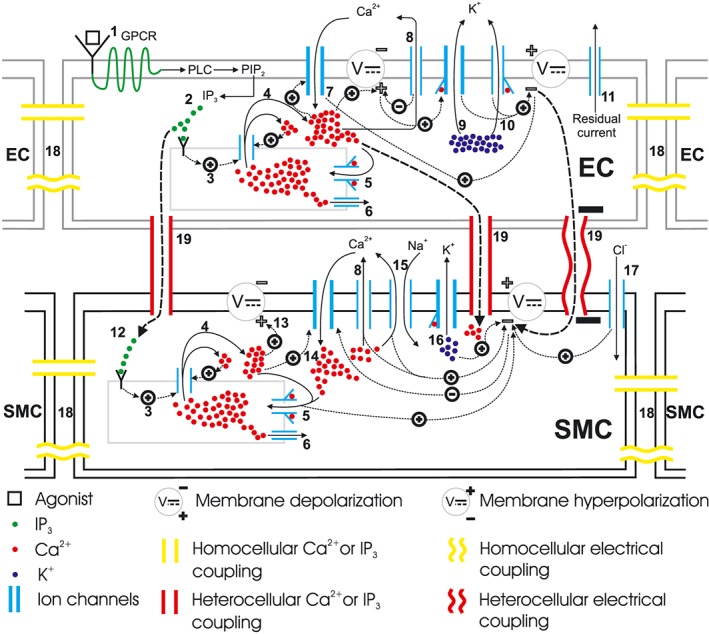
Schematic representation of mass transfer dynamics in a single endothelial cell (EC)/smooth muscle cell (SMC) unit; this model accounts for the essential mechanisms of IP^3^‐induced cytsolic Ca^2+^ release and the cascade of events following it. The diagram is adapted from the previous work by 27. The numbers refer to the pathways described in the text.

The adhesion of agonist (ATP) to the endothelial surface starts a complex cascade of reactions in the ECs and SMCs as shown in Figure 1. The following is a brief description of participating pathways:

Agonist binds to the purinergic (P2Y) receptors on the EC surface, activating the GPCR which then activates membrane‐bound phospholipase C (PLC). PLC activation allows phosphorylation of PIP2.
PIP2 gives rise to IP^3^ that is then released in the intracellular space. This nascent IP^3^ binds to IP^3^ receptor (IP^3^) on the ER/SR surface.
IP^3^‐bound IP^3^ induces release of Ca^2+^ ions from the ER/SR into the cytosol.
The Ca^2+^ release from intracellular store sensitises the IP^3^ further, which releases more Ca^2+^, thus making a Ca^2+^‐rich domain in the cytosol in both EC and SMC. The excess of intracellular Ca^2+^ depolarises the membrane potential.
ER/SR has low‐affinity binding sites on the cytosolic side of a channel, which constitutes a pump called the (SERCA) pump. Cytosolic Ca^2+^ encourages the replenishment of the intracellular stores via this pathway.
Ca^2+^ leaks from ER/SR consistently under a concentration gradient between cytosolic and ER/SR luminal Ca^2+^ and keeps the Ca^2+^ in equilibrium during a non‐stimulated state of the cell.
In an EC, the cytosolic Ca^2+^ favours the influx of extracellular Ca^2+^ from non‐selective cation channels.
The SERCA pump pushes out cytosolic Ca^2+^ to extracellular space.
10) In ECs, activation of KCa, upon binding to Ca^2+^ ions intracellularly at BKCa and SKCa, let K^+^ ions move out of the cytosol. This hyperpolarises the membrane potential.
Although K^+^ ions efflux is the main repolarising current, residual current (mainly consisting of monovalent ions) also contributes to membrane potential repolarisation.
The IP^3^ concentration increases in SMC cytosol via transmission of IP^3^ from coupled EC. This IP^3^ increase activates the downstream IP^3^‐induced Ca^2+^ release.
The IP^3^ induced, and calcium‐induced calcium release (CICR) Ca^2+^ depolarises the membrane potential.
The membrane depolarisation results in the influx of Ca^2+^ from VOCCs, which will close upon repolarisation in the following steps.
Ca^2+^, in addition to other pathways, is pushed out via Na/Ca exchanger.
Binding of Ca^2+^ ions to KCa opens BKCa channels in SMC causing K ions efflux and membrane repolarisation.
Influx of Cl_2_ ions add to the repolarisation.
Medium for intercellular communication via homocellular gap junctions can either be Ca^2+^, IP^3^ or membrane potential coupling.
Heterocellular gap junctions can couple an EC and an SMC via Ca^2+^, IP^3^ or membrane potential coupling. Hyperpolarised EC membrane potential can hyperpolarise SMC plasma membrane and consequently close VOCCs.

**Figure 2:**
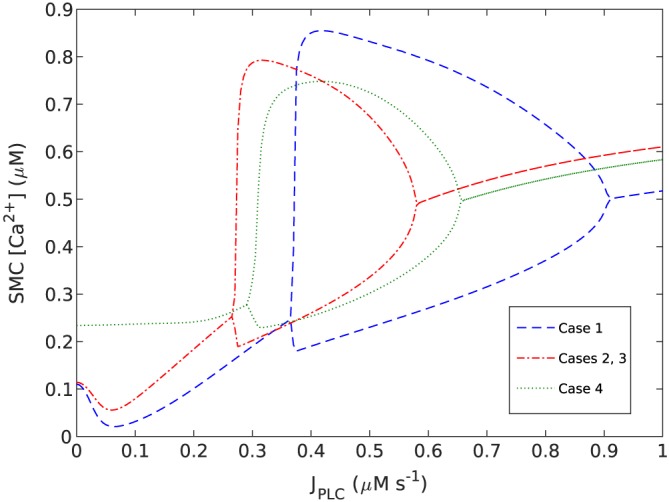
Bifurcation diagram for cytosolic smooth muscle cell (SMC) Ca^2+^ concentration in a single coupled endothelial cell/smooth muscle cell (SMC) unit as function of increasing J_*PLC*_ at the endothelial cell surface for coupling cases 1–4.

The ATP to J_*PLC*_ conversion models reported by 28 and 29 show that the relationship between ATP and IP^3^ is monotonic and increasing; thus it can be approximated by a linear function over the range of ATP. Therefore, a linear function is utilised to simulate the resulting relationship between ATP and the flux of IP^3^ into the EC, that is J_*PLC*_. The range of ATP concentration is represented by the equivalent values of the J_*PLC*_; the flux of IP^3^ into the EC is determined by the concentration of ATP in the vessel lumen. The whole range of J_*PLC*_ consists of three bands, which correspond to the ATP‐dependent Ca^2+^ dynamics in an SMC. As shown in Figure 2, low and high bands of J_*PLC*_ result in steady state Ca^2+^ concentrations, while the middle band shows the presence of Ca^2+^ oscillations. The oscillation envelope for this band indicates the relative high and low amplitudes for lower and higher J_*PLC*_ concentrations.

### 2.2 Cell coupling variations

The variations of coupling parameters described here model the connexin gap junction permeability used in the simulations in this work. The Cx37 and Cx40 down‐regulation along with Cx43 up‐regulation in early atheroma has been reported in the work of 30. 31 have shown that Cx43 gap junctions have a conductance change of 50 nS for each mV change in membrane potential; this value is used for modelling heterocellular electrical coupling. Homocellular coupling parameter values were obtained from the work of 32 to set electrical resistance at 33 MΩ(equivalent to 30 nS) as chosen in the work by 18. With a membrane capacitance between ECs of 30 pF whilst for SMCs this is 10 pF and using the results of 33. The macroscopic gap junctional resistance of 90 MΩ(equivalent to 11 nS) 33 is the value of the homocellular coupling for both EC and SMC set as 1000 *s* ^−1^. The Ca^2+^ and IP^3^ homocellular coupling parameters are treated as free in a similar way to the work of 18, assuming they are relatively weak; the value of is set to 0.05 *s* ^−1^. The coupling of membrane potential is a combination of simple ion diffusion across the gap junction and *drift* given by the product of ion concentration and spatial gradient of the membrane potential. For this particular model, the electro‐diffusion drift is ignored as it has been shown to have negligible effect. Unpublished work with coupled cellular oscillatory models shows this to be the case.

Table 1 summarises the coupling variations that have been used in the current work; the coupling parameter sets reproduce the parameters used in 27 in order to produce a comparison with the original 1D model with a 2D surface simulation. Cases 1 and 2 reflect ‘healthy’ (non‐pathological) coupling cases, where Case 2 has the added heterocellular Ca^2+^ coupling. The remaining two coupling configurations are designed to simulate pathological cases. In Case 3, *I* *P*_3_ coupling is added to the default homocellular EC coupling to simulate upregulation of Cx43 in lesion‐prone areas described in the work of 30. Case 4 has the homocellular EC and heterocellular membrane potential Ca^2+^ coupling disabled to simulate the coupling in progressive atherosclerosis when Cx43 connexin is the dominant mechanism of intercellular communication because of the down regulation of Cx37 and Cx40 connexins 5. It is recognised that these four cases represent simple approximations to a complex physiological phenomena and should not be seen as a complete description of the mechanisms involved. However, they are put forward an initial framework from which further more complex pathways can be simulated. In particular, the role of IP^3^ diffusion will need careful consideration due in part to its assumed smaller connexin channel permeability.

**Table 1:** Coupling cases defining healthy and pathological modes of intercellular communication.

	Homocellular		Heterocellular	
Number	SMC	EC	SMC ↔ EC	Case Description
1	V, Ca^2+^, *I* *P*_3_	V, Ca^2+^	V, *I* *P*_3_	Non‐pathological coupling.
2	V, Ca^2+^, *I* *P*_3_	V, Ca^2+^	V, *I* *P*_3_, Ca^2+^	Non‐pathological coupling case with the
				added heterocellular Ca^2+^ coupling.
3	V, Ca^2+^, *I* *P*_3_	V, Ca^2+^, *I* *P*_3_	V, *I* *P*_3_, Ca^2+^	Pathological case simulating lesion‐
				prone areas with the added *I* *P*_3_ coupling
				in ECs.
4	V, Ca^2+^, *I* *P*_3_	*I* *P*_3_	*I* *P*_3_	Pathological case simulating progressive
				atherosclerosis by removing homocellular
				(ECs) and heterocellular (SMCs
				and ECs) membrane potential and Ca^2+^
				coupling.

Figure 2 shows the bifurcation diagrams (maximum/minimum Ca^2+^ concentration vs the IP^3^ flux parameter J_*PLC*_); for case 1, the range of IP^3^ where oscillation exists is substantial whereas case 4 has a reduced domain, and the lower bifurcation points are shifted from approximately 3.7 to 3.0. Cases 2 and 3 are identical in their bifurcation points and maximum/minimum values of Ca^2+^ oscillations, because of the identical heterocellular coupling parameters. The bifurcation diagrams were generated for a single EC/SMC unit without homocellular coupling.

### 2.3 Computational solution methodology

Simulations of cellular dynamics even for small vessel segments require a substantial amount of computational resources because of the density and number of coupled cells comprising the endothelial and smooth muscle surfaces making up the arterial wall. The primary requirement in the development of the computation solution for the large‐scale simulations was the decomposition of the coupled EC and SMC layers into a number of ODEs systems, each corresponding to a number of ‘neighbourhoods’ of coupled cells grouped into quadrilateral surface elements later mapped to distinct computational domains. With a suitably chosen granularity of the surface‐to‐domain decomposition, the entire bifurcation surface can be mapped onto individual computational nodes in a parallel architecture.

#### 2.3.1 Bifurcation meshes

A method for generic parameterisation of bifurcating structures published by 34 offers an elegant solution for generating high‐quality surface meshes from centre lines of segmented arteries. For the presented cases, a simple symmetric bifurcation is used and is defined by three centre lines forming a Y‐shaped bifurcation. For the present model, we do not utilise a patient‐specific geometry but assume that the topological equivalent of an arterial bifurcation provides a sufficient surface.

**Figure 3:**
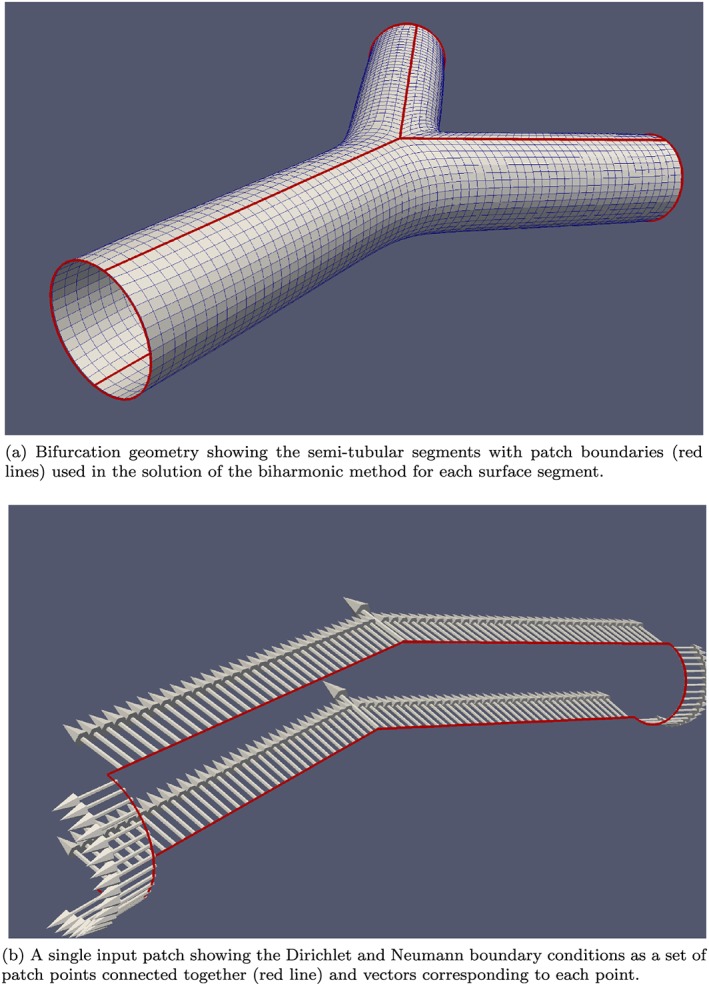
Composition of a bifurcations surface from semi‐tubular segments (top) with a single patch boundary conditions for a semi‐tubular segment (bottom).

Each Y bifurcation can be decomposed into three semi‐tubular surface segments by using the solution to a biharmonic equation, originally developed as a computer‐aided design tool by Bloor and Wilson 35. The biharmonic equation has the operator form given by

(1) $${\left[\frac{\partial^{2}}{\partial u^{2}}+a^{2}\frac{\partial^{2}}{\partial v^{2}}\right]}^{2}\phi =f(u,v),$$

where *ϕ* is the space vector (*x*,*y*,*z*) and *f*(*u*,*v*) can be either zero or some *a priori* defined function. For the present, we use *f*(*u*,*v*) = 0, where *u* and *v* are orthogonal coordinates, which provide axial and circumferential directions. The Neumann and Dirichlet boundary conditions for Eq. (1) define the rate at which the surface moves away from the boundary and its direction, respectively. Eq. (1) is discretised into *m* (*u* direction) and *n* (*v* direction) points on the boundary. With this method, the surface of each segment is unwrapped into a 2D *m* × *n* grid in the *u* and *v* directions, respectively; in this case, *m* and *n* represent the longitudinal and circumferential number of quadrilaterals in each semi‐tubular segment accordingly. The solution now becomes one of a 2D solution, rather than a 3D solution. To obtain a complete 3D surface, the semi‐tubular segments can be joined together at the boundaries. Figure 3(a) shows the boundaries of the semi‐tubular segments comprising the full bifurcation geometry. Each segment surface here is defined by the solution of the biharmonic equation. Figure 3(b) shows the Dirichlet and Neumann boundary conditions used as input for a single biharmonic solution.

The mapping of bifurcations to 2D‐quadrilateral meshes enables the decomposition of the whole computational domain into local domains, where each domain corresponds to a single computational task in a parallel environment. Furthermore, this type of decomposition enables the use of MPI‐specific virtual Cartesian topologies for efficient inter‐processor communication. Each tubular segment in the computational solution presented here is mapped to a 2D Cartesian grid of size *m* × *n* with a periodic boundary condition imposed along the longitudinal edge. In this case, the periodic boundary condition refers to the MPI‐enabled communication‐specific mapping of MPI tasks. It must be noted that the bifurcation surfaces are not constrained to symmetric or circular geometries as the solution of the biharmonic equation can be defined for any (continuous) boundary in (*x*,*y*,*z*) space.

**Figure 4:**
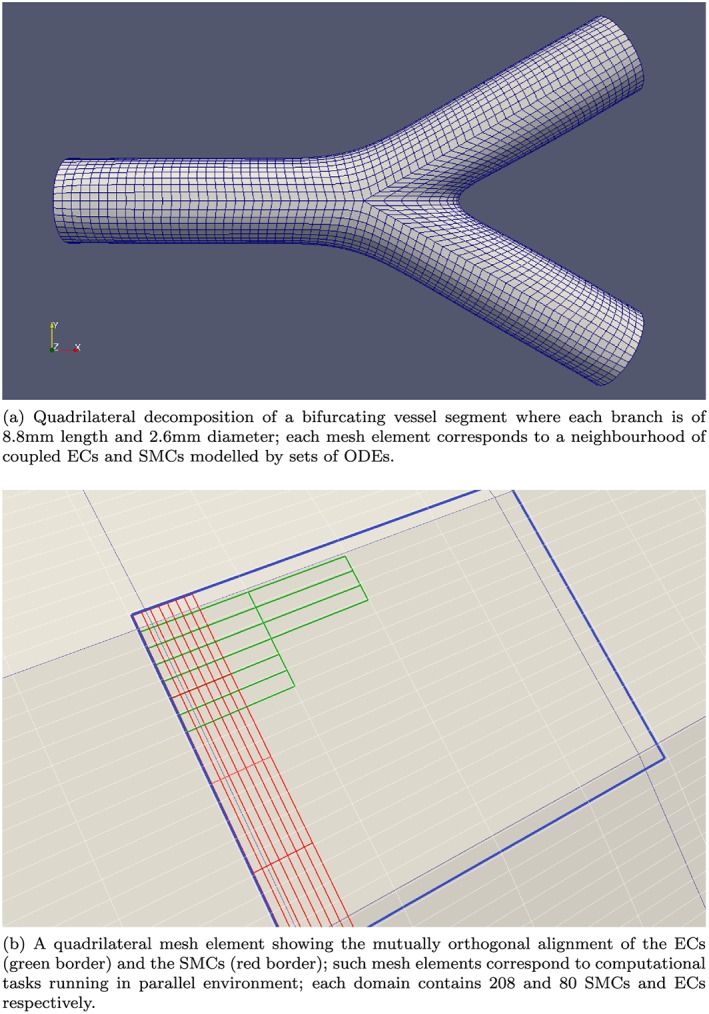
An example of a bifurcation surface mesh decomposed into *Q* = 4080 computational domain (top); arrangement of smooth muscle cells (SMCs) and endothelial cells (ECs) in a single quadrilateral mapped to a computational task in parallel environment (bottom). ODE, ordinary differential equation.

Figure 4(a) shows a bifurcation surface mesh consisting of *Q* = 4080 quadrilateral elements, where each element represents a cell neighbourhood on the bifurcation surface. With this type of surface decomposition, each quadrilateral domain corresponds to a parallel computational task running on a single core. The SMCs and ECs within each domain can then be placed into the quadrilaterals with little complexity because the (*u*,*v*) directions provide physiologically correct axial and circumferential alignments for ECs and SMCs, respectively. The SMC and EC cells are aligned orthogonally with respect to each other where ECs align axially with the flow, whilst SMCs align circumferentially. Figure 4(b) shows ECs and SMCs arranged in two layers within a single quadrilateral on the bifurcation surface. With the approximate physical dimensions of SMCs and ECs, 50 × 5*μ* *m* and 65 × 10*μ* *m*, respectively, the SMCs and ECs are arranged into ‘fundamental’ surface units, where the area covered by five ECs corresponds to the area of 13 SMCs; this arrangement corresponds to the layer arrangement used by 27. The surface units are placed into the quadrilaterals in a 4 × 4 grid giving 288 cells per a single quadrilateral/domain with 208 and 80 SMCs and ECs, respectively.

An implementation of the biharmonic equation solver in Fortran is freely available through online resources ‡Bjorstad, P. E. (1980). Numerical Solution of the Biharmonic Equation. Retrieved from http://http://www.netlib.no/netlib/bihar/dbihar.f.. The Fortran code was integrated into a C++ ‘wrapper’ as a part of a small library for generating surfaces from vessel centre lines; the library is using the relevant functionality of the (VTK) library 36 such as input, conversion of point clouds returned by the dbihar solver into surface meshes and output. The wrapper was implemented as a VTK‐style filter to be used as a part of a pipeline for generating meshes from vessel centrelines.

Two synthetic arterial bifurcation surface meshes were generated for the simulations reported in this work. The geometric configuration properties, such as proportional length and angle of the branches, were similar in both cases; however, the second mesh was generated for the vessel surface approximately twice the size of the first mesh. The physical dimensions of the cells are only relevant when generating the surface from a centreline with a specified vessel diameter; the knowledge of the physical dimensions of cells enables the generation of meshes with the correct cell density for a given centreline and vessel diameter. For example, a surface mesh for a bifurcation vessel segment with a parent and two branch arteries of 8.8 mm length and 2.6 mm diameter each is covered by about 1.2 million cells, where there are 326400 ECs, 848640 SMCs, with a total of 1175040 cells. If each EC and SMC are modelled by four and five ODEs, respectively, then the total number of ODEs for this mesh is 10575360, while the larger mesh requires 20901888 ODEs. These numbers of ODEs need to be solved thousands of times even for a short temporal domain. Table 2 provides the details of the bifurcation meshes.

**Table 2:** Parameters of the arterial surface meshes

Number	*L*_*B*_	*L*_*Max*_	*D*	*α*_*B*_	Q	ECs	SMCs	Total
1	8.84	17.68	2.55	30	4,080	326,400	848,640	1,175,040
2	10.92	21.84	4.08	30	8,064	645,120	1,677,312	2,322,432

Arterial surface meshes include branch length *L*_*B*_ (mm), maximum length *L*_*Max*_ (mm), diameter *D* (mm), branch centreline angle *α*_*B*_ (degrees), number of domains *Q*, number of ECs, number of SMCs and the total number of cells in a vessel bifurcation segment; each domain for both mesh variants included a total of 288 cells, with 208 and 80 SMCs and ECs, respectively.

#### 2.3.2 Parallel simulation implementation

The simulations were performed on the IBM BlueGene P architecture at the University of Canterbury High Performance Computing Centre. The implementation also has been tested on the BlueGene Q architecture at the Argonne National Laboratory.

For each quadrilateral domain, the system of ODEs was solved using an explicit Runge Kutta (4, 5) scheme initially implemented in Fortran as rksuite_90 component of the (NAG) Fortran Library 37; in the work reported here, a version of rksuite_90 converted to C++ was used instead. At regular time intervals, the state variables from the cells along the edges of the quadrilateral domains were passed to the adjacent cells in the neighbouring domains. The inter‐domain communication was implemented through the MPI library. The solution for inter‐domain communication and the corresponding boundary conditions enforcement within a single MPI domain was implemented following the mesh ghost‐cell communication pattern recommended for discretised domain decomposition 38. For the simulations presented here, the inter‐domain communication interval was set to 0.01 s; this interval was chosen to maintain the balance between MPI communication and computation times.

A parallel simulation requires as input the basic geometric configuration the surface for a given bifurcation in terms of the number of quads along axial and circumferential dimension; in addition, each simulation requires the configuration of the coupled EC and SMC in a quad. The quads are grouped into 2D Cartesian meshes, each representing a tubular segment/branch of a bifurcation. Within each 2D Cartesian mesh, the quads correspond to computational domains, which are mapped to distinct MPI tasks. In addition, each simulation requires an explicit agonist (ATP) map, where each EC cell is assigned a surface layer ATP value. The agonist maps were generated from the corresponding EC surfaces in order to preserve the implicit topology of the cells. The types of agonist maps used in this work are described in Sections 2.4 and 2.5.

The state variables' values for ECs and SMCs were written to HDF5 format after every elapsed physiological second. In the post‐processing step, the HDF5 output data for every second was mapped onto the surface/cell mesh and written out in VTK's VTU format, which is an XML‐based format with binary data for unstructured grid data. The visualisation and analysis were performed with the ParaView 39.

### 2.4 Synthetic agonist map

Work by 14 has shown that even in a time‐dependent CFD‐based solution, the ATP concentration can be adequately modelled by a time‐averaged profile in areas exhibiting flow separation and low WSS. The areas characterised by large Péclet number (Pe) values (defined by the low diffusion coefficient of ATP) and thick concentration boundary layer of mass transfer are not significantly affected by the pulsatile flow. The ATP concentration in these areas can be represented by functions of a linear form. In addition, these areas are often preceded (upstream) and followed (downstream) by a constant WSS profile 14.

**Figure 5:**
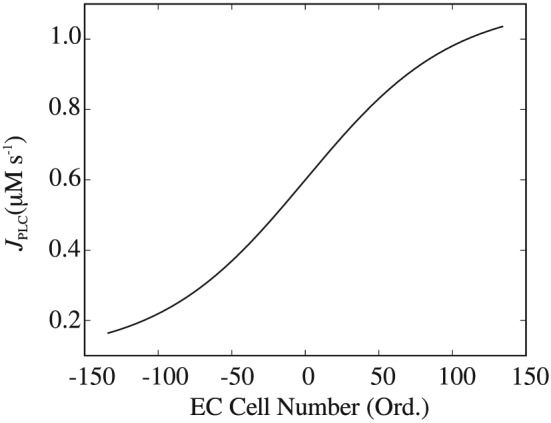
Sigmoid function used in the modelling of the adenosine triphosphate distribution for a synthetic agonist map; adenosine triphosphate concentration is linearly related to the rate of inositol trisphosphate (IP^3^) production given as J_*PLC*_; the function is centred on the bifurcation point; the negative endothelial cell positions are decreasing towards the inlet of the bifurcation segment, while the positive numbers are increasing towards the outlets. EC, endothelial cell.

**Figure 6:**
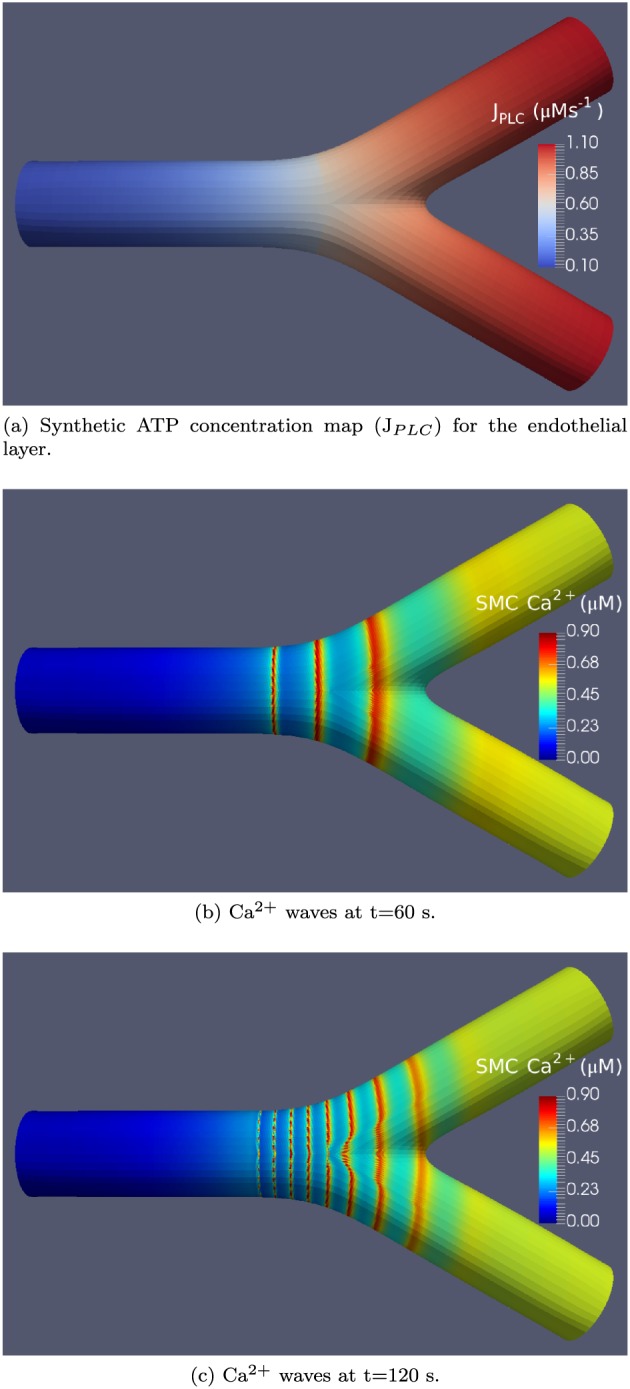
Synthetic adenosine triphosphate (ATP) concentration map and a visualisation of Ca^2+^ waves for the *Q* = 4080 mesh under Case 1 coupling conditions.

Synthetic agonist maps for the simulations presented here were generated with a sigmoid‐like distribution of J_*PLC*_ values in the endothelial layer defined as a function of axial distance only. As noted previously, ATP concentration is linearly related to the rate of IP^3^ production given as J_*PLC*_. Figure 5 shows plot of a sigmoid function for a surface mesh in Figure 4(a). The properties of a synthetic agonist map generated in this manner enable a simulation for an arterial bifurcation where there exist both constant and linearly‐varying ATP concentrations. Figure 6(a) shows the corresponding synthetic ATP map for number of domains *Q* = 4080.

### 2.5 CFD‐based (physiological) agonist map

The CFD simulations for generating physiologically realistic ATP maps follow the algorithm described in the work of 14. Flow and mass transfer fields were solved with the open‐source CFD tool, OpenFOAM 40. The flow field is solved iteratively via the continuity and momentum Eqs. (2) and (3), on the assumption that the blood flow is steady state, incompressible and of Newtonian rheology:

(2) ∇·u=0

(3) $$u\cdot \nabla u=\nu \nabla^{2}u-\nabla p.$$

Here, **u** represents the blood velocity vector field **u** = [*u*,*v*,*w*], *p* the kinematic pressure field and *ν* the kinematic viscosity. The species mass transport was solved simultaneously via the conservation of species equation given by

(4) $$u\cdot \nabla \phi =D\nabla^{2}\phi ,$$

where *ϕ* is the species ATP concentration and *D* is the isotropic diffusion coefficient. The walls of the artery are assumed to be rigid and stationary. Due to the very high Péclet number, the time‐averaged nucleotide concentration does not differ significantly from the steady state concentration 14. The governing equations were solved iteratively via the Semi‐Implicit Method for Pressure‐Linked Equations algorithm for pressure‐velocity coupling; the convective terms were handled with second‐order upwinding for momentum and third‐order upwinding Monotonic Upstream‐Centred Scheme for Conservation Laws scheme for chemical species.

A parabolic Dirichlet velocity profile was implemented on the inlet (the peak velocity was determined for the actual inlet area and the specified flow‐rate) and zero velocity gradient Neumann boundary conditions on the outlets. The pressure field values were specified as zero‐pressure gradient Neumann boundary conditions for the inlet and zero‐pressure Dirichlet boundary condition on the outlets.

For the species boundary conditions, a constant Dirichlet value of 0.1 *μ* *M* was specified on the inlet and zero gradient Neumann boundary conditions on the outlets. As noted in Section 2.1, the relationship between ATP and IP^3^ production rate is monotonic and increasing, which can be approximated by a linear function. Therefore, a linear relationship between ATP and the IP^3^ flux into the EC (*J*_*PLC*_) is deemed sufficient. Following the work of 17, a boundary condition is defined as follows:

(5) $$D\frac{\partial \phi (x,0)}{\mathrm{\partial y}}=\mathrm{K\phi}(x,0)-S(x)$$

Here, *S*(*x*) represents the production of ATP as a function of WSS in the form of:

(6) $$S(x)=\frac{\tau_{w}(x)}{\tau_{m}},$$

where the *K* and *τ*_*m*_ values as reported in the work of 17. In this case, the boundary condition on the endothelial surface is set to Robin (reactive) boundary condition specified on the arterial walls. This boundary condition is implemented iteratively by specifying a Neumann boundary condition for the ATP gradient, where the gradient itself is a function of ATP concentration. For every iteration, the boundary condition is evaluated based on the current ATP concentration, then the ATP gradient is specified on the wall, and the scalar transport equation is solved. The process is repeated until convergence is obtained.

All other parameters for mass transport were taken from the work of 17, and the WSS is calculated from the Navier–Stokes flow‐field. Michaels–Menten formulation used to determine the IP^3^ concentration in the EC. Figure 11(a) shows the ATP concentration, resulting from CFD mass transport simulations for a mesh with the number of domains *Q* = 4080.

## 3 Results

The simulations were performed with both synthetic and CFD‐based ATP agonist surface concentration profiles. In both cases, the simulations were carried out for all four cell‐coupling cases. All simulation runs were executed for the *Q* = 4080 mesh (corresponding to 4080 cores of Blue Gene/P). For the *Q* = 8064 mesh (corresponding to 8064 cores of Blue Gene/P), only Case 1 coupling with a synthetic agonist profile was performed. This simulation was designed to verify that a larger population of cells did not change the dynamics of the simulations in a significant way. All simulations were run for 1000 physiological seconds, where output was recorded after every physiological second. The small mesh bifurcation simulations with *Q* = 4080 cores on Blue Gene/L on average took approximately 20.3 h to complete, while the large mesh bifurcation simulations with *Q* = 8064 cores on Blue Gene/P took approximately 15.1 h. The visualisations for all simulations are available on the UC HPC YouTube Channel.

### 3.1 Synthetic agonist map

Figure 6 shows the agonist map for the *Q* = 4080 mesh along with the propagating Ca^2+^ oscillations in the SMC layer at two simulation steps of t=60 and 120 s, respectively. The oscillations originate in the downstream branches of the bifurcation at the upper end of the J_*PLC*_‐dependent Ca^2+^ oscillatory domain §In the simulation snapshots the ‘upstream’ direction is defined from right to left (in opposition to blood flow) and ‘downstream’ to be from left to right (in the same direction as physiological blood flow).. As the oscillations propagate upstream, the wave amplitude increases, while the wave period decreases. This behaviour continues with the time progress of the simulation.

**Figure 7:**
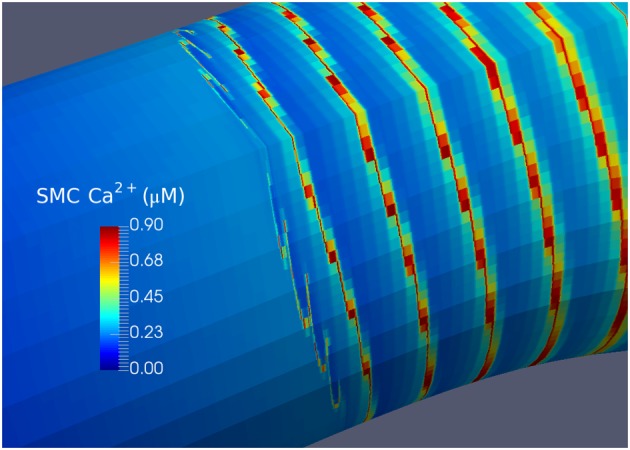
Propagating wave‐front at *t* = 150 s for coupling Case 1 with the 4K mesh. SMC, smooth muscle cell.

Figure 7 zooms in on the upstream wave front, where the oscillations are dampened while propagating into the non‐oscillatory domain at t=150 s. The phenomenon of wave propagation past the oscillatory domain is known to exist in excitable media. In this case, the Ca^2+^ waves travel past the oscillatory domain because the system is excitable for a range of low J_*PLC*_ in the non‐oscillatory domain close to the Hopf bifurcation. This excitability is found in similar Ca^2+^ SMC models such as the model by 41. Spiral waves can form in excitable systems from open wave ends 42, which we see in Figure 7.

**Figure 8:**
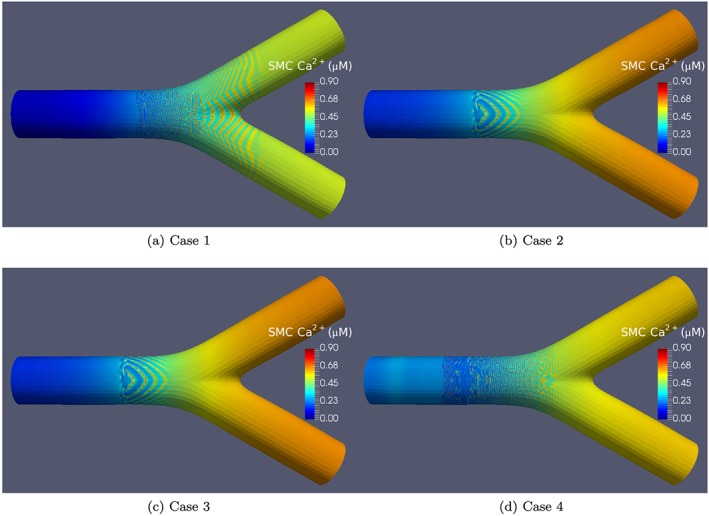
Wave plots for the synthetic agonist map for all coupling cases at *t* = 600. SMC, smooth muscle cell.

Figure 8 shows the comparative visualisations for all coupling cases at the later stage of the simulations (t=600 s). This illustrates the differences in the oscillatory behaviour determined by the differing coupling configuration.

In order to generate 2D space/time plots for visualising and comparing temporal wave propagation patterns, a subset of SMCs was extracted from all time steps for a given simulation. This subset was defined by plotting time‐dependent SMC concentrations along a line extending along the outer surface of the bifurcation from inlet to outlet.

**Figure 9:**
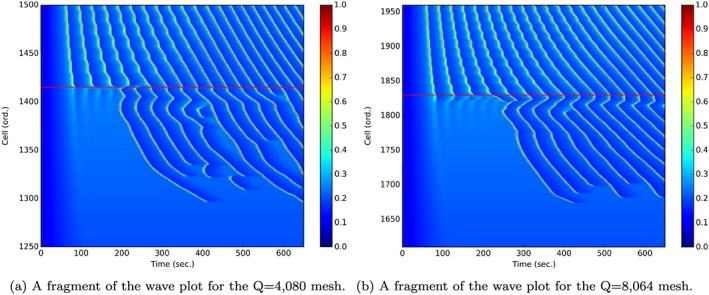
Comparison of the Ca^2+^ (*μ* *M*) waves for Case 1 coupling in the SMC, smooth muscle cell layer along the extracted SMC, smooth muscle cells line; the red line indicates the lower bound of the J_*PLC*_‐dependent Ca^2+^ oscillatory range, where the oscillations are ‘extinguished’ at the start of the simulation, as the simulation progresses Ca^2+^ waves tend to spread into the non‐oscillatory domain.

Figure 9 presents the comparison of the Ca^2+^ oscillation dynamics for the two mesh sizes. The 2D wave plots show that the overall simulation results show significant similarities for both bifurcation meshes. These differences are explained by the following:

1. The *Q* = 8064 mesh was not an exact scaling of the *Q* = 4080 mesh.
2. The Hopf bifurcation point (lower bound of oscillations for a single SMC) in the ATP profile for the *Q* = 8064 bifurcation is at a different location in the parent artery segment compared with the *Q* = 4080 mesh.

The Ca^2+^ oscillations in the SMC under the Case 1 coupling conditions are initially ‘extinguished’ at the lower border of the oscillatory J_*PLC*_ concentration range. However, in both cases the oscillations propagate upstream past the lower boundary (red horizontal line in the figure) at approximately 200 and 250 s for the *Q* = 4080 and *Q* = 8064 meshes, respectively.

**Figure 10:**
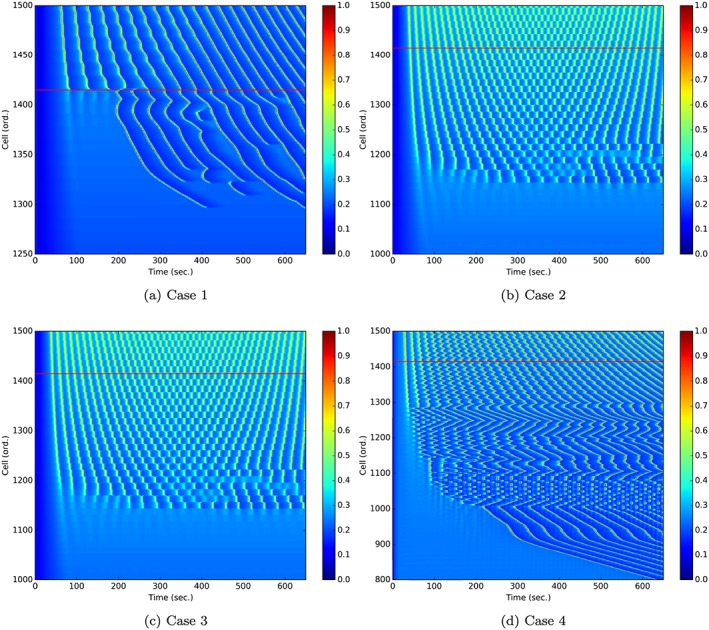
Comparison of zoomed‐in plots showing the Ca^2+^ (*μ* *M*) waves in the SMC, smooth muscle cell layer along the extracted SMCs line; the red line indicates the lower bound of the J_*PLC*_‐dependent Ca^2+^ oscillatory range, shown originally in Figure 2.

Figure 10 shows the zoomed‐in versions of the space time plots for all four coupling cases. Significant differences exist for the wave profiles in each of the cases. The visualisations for all four coupling cases with the synthetic ATP map are available online ¶Coupling Case 1—https://www.youtube.com/watch?v=ax8I6rZG-6Q,Coupling Case 2—https://www.youtube.com/watch?v=HfMHsZrM8bw,Coupling Case 3—https://www.youtube.com/watch?v=lx-Pza_{Z}HVk,Coupling Case 4—https://www.youtube.com/watch?v=5JpfDcs5GTA..

### 3.2 Physiological agonist map

**Figure 11:**
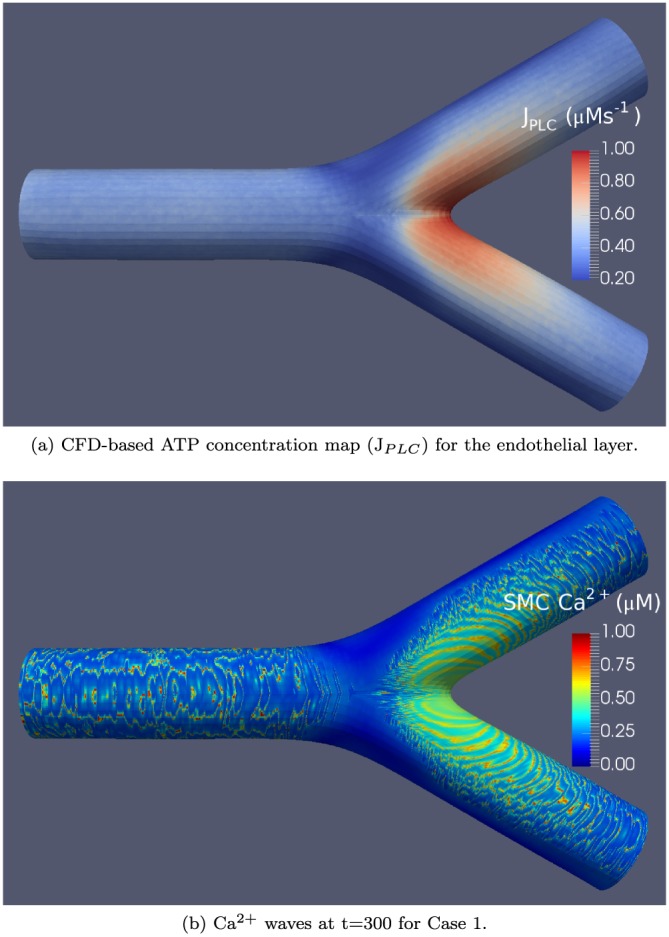
CFD‐based adenosine triphosphate concentration map and a visualisation of Ca^2+^ waves.

CFD‐based agonist maps were generated only for the *Q* = 4080 bifurcation surface mesh described in Section 2.3.1. Figure 11 shows the agonist map for this surface along with the propagating Ca^2+^ oscillations in the SMC layer at time *t* = 300 s. The oscillations originate largely in the branches of the bifurcation where the J_*PLC*_ concentration is high compared with the rest of the vessel segment.

**Figure 12:**
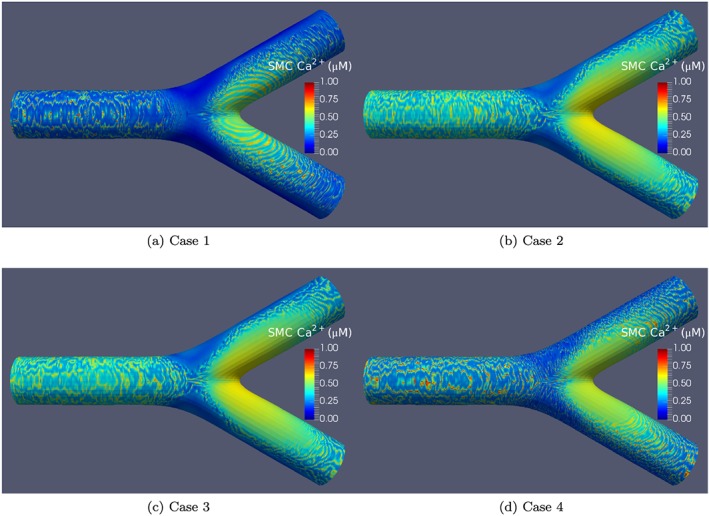
Visualisation of Ca^2+^ oscillations for all four coupling cases for the computational fluid dynamics‐based agonist map at time step *t* = 300.

Figure 12 presents a comparison of simulation results with a CFD‐based agonist map for all four coupling cases at *t* = 300 s. One commonality between all cases is that similar to the synthetic agonist map the oscillations propagate along the direction of the decreasing ATP gradient. Although not shown in the static figures, waves propagating on the surface of the inlet cylinder seem to do so in a circumferential direction. Variations in Ca^2+^ are determined from the small neighbourhood of the lower bound of the J_*PLC*_‐dependent Ca^2+^ oscillatory range, as shown in Figure 2. The wave trains seen in the downstream branches move from right to left, along the gradient from high to low ATP concentration. For Case 1, oscillations occur at the inner surface of the bifurcation point whilst for all of the other three cases, this area is devoid of oscillations and holds a constant Ca^2+^ concentration. The visualisations for all four coupling cases with the CFD‐based ATP map are available online ∥Coupling Case 1—https://www.youtube.com/watch?v=UPZZdbF8De0,Coupling Case 2—https://www.youtube.com/watch?v=QH9gyH5s1fo,Coupling Case 3—https://www.youtube.com/watch?v=yj_{q}1QIbrdg,Coupling Case 4—https://www.youtube.com/watch?v=8q6hrFsUy4s..

### 3.3 Parallel scalability

**Figure 13:**
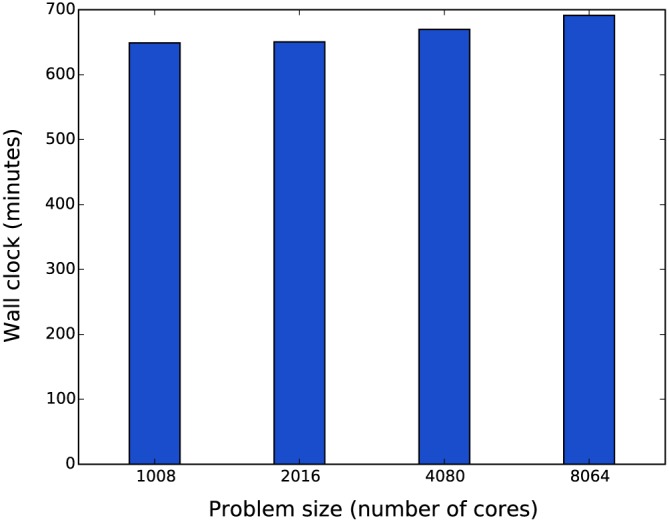
Weak scaling results for four meshes of increasing size (1000 physiological seconds simulations).

**Figure 14:**
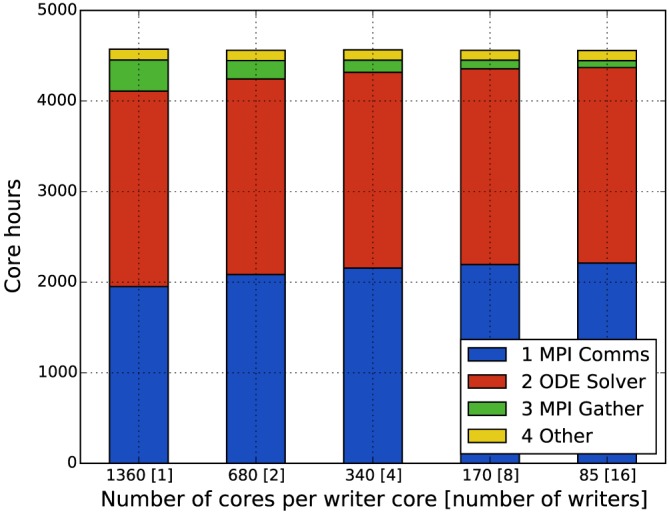
Simulation time breakdown for variable number of IO‐dedicated cores (1000 physiological seconds simulations); HDF5 output time is not shown due to negligible values. MPI, message passing interface; ODE, ordinary differential equation.

Figure 13 shows weak scaling results for four meshes of increasing size. Each simulation was performed for 1000 physiological seconds, which on average took 6.5 h. The results show a remarkable parallel scalability of the simulations with the increasing total problem size, while keeping the individual core workload constant. Figure 14 shows the breakdown of core hours between intra‐domain ODE solver time, inter‐domain MPI communication time and IO‐specific MPI aggregation time. This figure shows that with the given configuration of simulations (the number of cells per MPI domain, inter‐domain communication interval and frequency of HDF5 IO) the IO‐specific MPI aggregation time is optimal for the balance between computation and communication for this specific type of simulations.

## 4 Discussion

Although the analysis of the model is qualitative in nature, the simulations results allow us to investigate the differences among coupling cases for a particular region in the ATP map. The visualisations (figures in the text as well as supplementary videos) are designed to demonstrate the emergent cell behaviour between healthy and pathological conditions. As it has been noted earlier, the four coupling cases represent simple approximations to a complex physiological phenomena and should not be seen as a complete description of the mechanisms involved. However, they are put forward as an initial framework from which further more complex pathways can be simulated. In particular, the role of IP^3^ diffusion will require a close review due in part to its relatively small homotypic connexin channel permeability even though Ca^2+^ buffering is taken into account.

### 4.1 Synthetic agonist map

We first turn to discussion and analysis of Ca^2+^ waves for the synthetic agonist concentration maps. Figure 6 for Case 1 shows clearly the propagation direction of waves and that the wave length is an increasing function of axial distance (length decreases as the wave moves from right to left). Initial analysis of the dynamics of the SMC indicates that this is due to reduced cytosolic Ca^2+^ whilst remaining in the oscillatory regime. Overall, waves do not propagate into areas of high ATP/J_*PLC*_/IP^3^ concentrations defined by the upper bound of the J_*PLC*_‐dependent Ca^2+^ oscillatory range shown in Figure 2.

Figure 7 indicates three characteristic features of the wave propagation phenomenon. Firstly, that of variations in the width of the propagating wave as a function of circumferential direction. This is due to diffusion in both directions (axial and circumferential) where as stated before the width is a function of the flux of Ca^2+^ moving into neighbouring cells and being either taken up by the SR or diffused out to other nearby cells whose Ca^2+^ concentration is of a size that will allow ions to move across the cell boundary through gap junctions. The second feature is that of how waves terminate: as the concentration of ions in the cells decreases due to diffusion, the waves become weak and narrow in width and eventually are extinguished by seemingly curving back upon themselves. Spiral waves are known to occur in a number of biological conditions as reported in the work of 42. Finally, the results show that the Ca^2+^ is dispersed in wave packets. The blocks of Ca^2+^ concentration are not single cells (because these are much smaller than a single mesh element). The initial examinations indicate that these packets of cells have similar cytosolic Ca^2+^ concentrations and are in phase in terms of their oscillatory dynamics. However, it is as yet unclear as to the exact reason for this.

Figure 8 indicates the variations in wave propagation phenomena as a function of the four cases of coupling. Case 1 (Figure 8(a)) has no heterocellular Ca^2+^ coupling. Thus, any oscillations produced in the ECs do not acquire fully propagated to the SMCs, although because of the heterocellular IP^3^ diffusion, cytosolic Ca^2+^ is propagated from cell to cell in only small amounts. It should be noted that if the sensitivity of the *I* *P*_3_ receptors on the SR is increased then this may very well produce profiles similar to Case 2 because of increased cytosolic Ca^2+^ effluxed from the SR.

For Case 1, waves occur for a substantial distance downstream (left to right) in the daughter artery whereas for Cases 2, 3 and 4, the daughter arteries are relatively free of oscillation. For Case 2, compared with Case 1, the dynamics are substantially different due to the inclusion of heterocellular Ca^2+^ coupling. The agonist‐induced increase in IP^3^ and subsequent Ca^2+^ efflux from the ER is now diffused to the SMC via gap junctions. The increase in SMC Ca^2+^ causes oscillations (via the CICR mechanism) to occur, and further diffusion allows cells not normally participating in an oscillatory regime to become activated. The curvature of the waves seen in (Figure 8(b)) is a result of a complex dynamic. Initially, the waves propagate from the bifurcation upstream in an axisymmetric fashion, similar to Case 1. When the waves are weakened close to being extinguished, a reflected wave allows Ca^2+^ waves to move upstream. The curvature of the waves changes from positive to negative as time progresses. This can be seen in the video associated with the supplementary videos provided online. Case 3 (Figure 8(c)) is similar to Case 2, as expected because the difference between Cases 2 and 3 is that EC homocellular IP^3^ coupling is removed in Case 3. It indicates once again that Ca^2+^ coupling is the dominant mechanism of wave propagation.

Case 4 coupling parameters results are shown in Figure 8(d); in this case, the wave dynamics are more chaotic and unstructured compared with the other coupling parameter cases. At the first stages of activation, the waves propagate upstream but become very short and rapidly disappear at a position just upstream from the Hopf bifurcation point denoted in Figure 2. As time progresses, the waves continue to move upstream and become extinguished further and further upstream from the original Hopf bifurcation point. At approximately *t* = 100 s, the upstream Ca^2+^ dynamics initiate very quick ‘flashes’ of intense concentration just downstream of the upstream wave domain border; these ‘flashes’ are evident for approximately 5 to 6 s and leave a band of inactive Ca^2+^ as shown in Figure 8(d) at *t* = 600 s. These can be seen in the supplementary video material provided online.

In the space/time concentration plots shown in Figure 9, the Ca^2+^ waves along the outer edge from inlet to upper daughter outlet indicate a strong similarity between the *Q* = 4080 (Figure 9(a)) simulation and the *Q* = 8096 simulation (Figure 9(b)). The smaller mesh is not an exact physical scaling from 4080 to 8064 mesh elements; thus, the J_*PLC*_ maps are slightly different for the two mesh sizes. However, in both meshes the waves initially stop at the Hopf bifurcation point. After approximately 200 s, the waves move upstream with varying wave speed. The figures show that waves stop and start suddenly. The analysis of 1D waves (data not shown) indicates that this is caused by the diffusion of Ca^2+^ to the extent where the cytosolic Ca^2+^ is too low to cause the CICR mechanism to initiate oscillations. A finite time is needed for diffusion to build up the cell Ca^2+^ again to renew oscillatory behaviour.

Figure 10 shows the space/time Ca^2+^ concentration plots for all four coupling cases. Case 1 coupling simulation results have been discussed earlier. Case 2 coupling plot indicates the effect of ‘wave packets’ propagating in both upstream and downstream directions. This points to distinct ‘communities’ of cells whose Ca^2+^ oscillations are in phase and hence do not provide sufficient diffusion between them. Only when the boundaries of these packets allow significant Ca^2+^ into the community does the cell packet breaks up. This phenomena need further analysis to confirm our initial explanation. Case 3, as mentioned previously is similar to Case 2. Case 4, however, presents a combination of phenomena where packets of cells exist throughout specific positions on the surface. At the later simulation stages, the waves propagate further upstream in a well‐defined manner. For Cases 2, 3 and 4, the propagation beyond the bifurcation point starts immediately in contrast to Case 1, where it takes a substantial time to produce upstream wave propagation past into the non‐oscillatory region. It is clear that the diffusion of Ca^2+^ through heterocellular gap junctions is the dominant mechanism for all wave profiles, although gap junction transmission of IP^3^ does have an effect through the action of IP^3^ receptors on the SR of an SMC. However, this may be a function of the *I* *P*3 permeability value being relatively small.

### 4.2 CFD‐based agonist map

We now turn to the case for the CFD‐based ATP agonist map. Figure 11 shows the ATP surface profile derived from the CFD solution described earlier. Here, we see a substantially different ATP concentration from the synthetic map. Figure 11(b) shows the Ca^2+^ concentration at time *t* = 300 s. Although this ATP map is substantially different from the synthetic condition, the waves still propagate in a direction towards low ATP (or J_*PLC*_ rate) concentration and, for this particular, ATP concentration profile waves move downstream into the daughter artery. Low ATP concentration occurs upstream in the parent artery segment. Here, complex waves and patterns occur, but this is due to the low levels of ATP.

### 4.3 Coupling and the transition from *healthy* to *diseased* conditions

A comparison with the simulation results for the CFD‐based ATP map in Figure 12 demonstrates that these cases have markedly different ATP‐defined oscillatory responses in the areas that are most likely to develop atherosclerotic plaque, that is the outer bends of a bifurcation surface used for the simulations in this work. The outer bends of the bifurcations under the assumed non‐pathological coupling conditions (Cases 1 and 2) demonstrate lack of oscillatory behaviour. Under the pathological coupling conditions (specifically Case 4) the cells in the outer bends demonstrate a markedly different behaviour. While the exact transition path from healthy to diseased conditions remains to be established, it is reasonable to hypothesise that in an *in vivo* domain localised cell‐coupling parameters could change in response to the haemodynamic‐dependent agonist concentrations and stress on the cyto‐skeleton over long periods of time. This agonist‐induced change in coupling conditions could be one of the triggers linked with the transition from healthy to diseased state. This hypothesis requires further investigation with more complex coupling models. It may be suggested that the transition from healthy to diseased state is a multi‐stage process where a response to the changing haemodynamic conditions results in coupling condition changes followed by the corresponding change in the oscillatory behaviour. Figure 12 illustrates this hypothesis. For example, the outer bend localised coupling could change over the course of time from healthy to pathological coupling, resulting in a different response to the agonist. For Case 1 (Figure 12), no oscillations occur in the outer bend (at any time during the simulation) whilst for Case 4, waves occur in the outer bend area for the majority of the simulation time, starting at approximately 100 s into the simulation. Further investigation of the response may help reveal the mechanics of arterial plaques growth.

### 4.4 Limitations and future work

Limitations of the model include only one‐cell‐deep layer of SMCs. Future work will extend the simulations to include a model of SMC layer of variable thickness in terms of the number of cells. Additionally, Figure 6(c) indicates that there is an apparent dependence of the wave speed (an increase) on the size of the quadrilaterals in the neighbourhood of the bifurcation. This is an artefact of the tessellation of the bifurcation surface. Around bifurcations, the area and dimensions of the surface quads are accurate only to the level of magnitude because of the complexity of the surface geometry. Some of the quads acquire stretched out along the first dimension and shortened along the second dimension. Thus, in this neighbourhood, the density of cells varies, because for each quadrilateral tessellating the arterial surface, there are a specific number of SMCs and ECs along each dimension. The simulation uses lumped parameter equations where the ion concentrations are constant throughout the cell. Consequently, waves appear to travel faster along the neck of the bifurcation. Although this is not physiologically accurate, diffusion inside the cell is ignored in the current model because of the assumptions of a lumped model. Future work will account for the improved distribution in cell size across the quadrilateral domains while allowing variations in ionic cytosolic concentrations. Most importantly, future projects will investigate atherosclerotic disease states transitions along with dynamic state transitions within homo‐typic and hetero‐typic coupling parameter space. This will involve explicit modelling of the effects of WSS on the endothelium as well as spatially varying coupling coefficients simulating the alignment of ECs in straight arteries compared with the random orientation and distribution of ECs found close to bifurcations and stagnation points defined by the blood fluid mechanics. Another branch of future work will extend the model to predict the sites of atherosclerosis initiation and compare the results of simulations to image‐based clinical data.

## 5 Conclusions

We have developed a massively parallel framework for performing 3D simulations of coupled EC/SMC dynamics in a bifurcating artery. The simulations reported in this work include up to 2.3 million cells modelled by more than 20 million ODEs. The results of the time‐dependent simulations show a radically varying range of Ca^2+^ wave propagation profiles determined by the coupling configurations. The simulations demonstrate that heterocellular diffusion of both IP^3^ and Ca^2+^ through gap junctions plays an important role in information transfer over large spatial scales. Zero membrane potential coupling does not eliminate Ca^2+^ oscillations but provides a significant difference in the oscillatory dynamics over scales much larger than a single cell. Indeed the simulations show that IP^3^ is an important transport mechanism for oscillations to occur and one that should not be neglected over and above that of Ca^2+^.

The results of the reported simulations show that for both the synthetic and CFD‐based agonist maps the phenomena of macro‐scale Ca^2+^ oscillation propagation is strongly dependent on the ATP gradient along the EC layer and the Ca^2+^ cellular dynamics. Although the bifurcation meshes used in this work are symmetric, and the use of symmetric boundary conditions could have been used to reduce the computation time, the simulations were performed with full bifurcation meshes in preparation for large‐scale simulations with meshes modelling realistic arterial segments where daughter arteries are of different diameters, and the bifurcation angles change depending on the location of the arterial segment in the human vasculature. We believe that the coupled EC/SMC dynamics modelling framework is an invaluable tool capable of providing deep insights into the cellular dynamics and the development of vascular disorders in connection with the local properties of arterial geometries. We would like to emphasise that the relationship between Ca^2+^ dynamics (in terms of cellular oscillations and wave propagation) and vascular disease is tentative at present, while considerable further research is required to advance the understanding of the interplay between vascular geometry, fluid dynamics and cellular function. It is our intention to extend the parallel implementation of the model to simulate patient‐derived geometry at a realistic scale. This work will involve the use of substantially larger computational resource. The presented framework is a foundation step towards simulating a full coronary tree, which involves the solution of billions of ODEs, and is considered to be a classic exascale problem.

## Acknowledgements

The authors would like to thank the members of the University of Canterbury High Performance Computing Centre, Tony Dale and François Bissey for their enthusiastic support and expert advice on the technical front. We also thank Allanah Kenny for the dynamical system behaviour analysis and the final manuscript review.

The authors wish to acknowledge the contribution of NeSI to the results of this research. New Zealand's national compute and analytics services and team are supported by the New Zealand eScience Infrastructure (NeSI) and funded jointly by NeSI's collaborator institutions and through the Ministry of Business, Innovation and Employment.

## Supporting information

Supporting info item

Supporting info item

## Appendix A

### A.1 Coupled cells physiological model

A single pair EC/SMC model from the work of 18 accounts for the essential mechanisms of IP^3^‐induced cytosolic calcium release and the cascade of the triggered processes. The work of 27 extends the single SMC and EC Koenigsberger model with the homocellular and heterocellular coupling. Similar to the intracellular ion concentrations and membrane voltage potential, the coupling terms are defined as ion fluxes; thus, the intercellular fluxes are added to the corresponding conservation equations for the intracellular concentrations and membrane voltage potential. The parameters of the EC/SMC model are provided in 27.

The following equations ((A1)—(A5)) specify the dynamics for cytosolic calcium concentration (*c*), SR calcium concentration (*s*), membrane potential (*v*), cytosolic IP^3^ concentration ( I) and the open state probability of the calcium‐activated potassium channels (*ω*) for the SMC layer.

(A1) $$\begin{array}{cc} \frac{\mathrm{dc}}{\mathrm{dt}}= & \;\;\underset{\text{Cytosolic}\;\mathrm{SMC}\;Ca^{2+}\;\text{terms}}{\underset{︸}{J_{IP_{3}}-J_{\text{SR\_}\text{uptake}}+J_{\text{CICR}}-J_{\text{eff}}+J_{\text{leak}}-J_{\text{VOCC}}+J_{\mathrm{Na}/\mathrm{Ca}}}}\\ +\underset{\text{Coupling}\;Ca^{2+}\;\text{terms}}{\underset{︸}{J_{\mathrm{Ca}}^{\mathrm{SMC}↔\mathrm{SMC}}+J_{\mathrm{Ca}}^{\mathrm{SMC}↔\mathrm{EC}}}} \end{array}$$

(A2) $$\frac{\mathrm{ds}}{\mathrm{dt}}=\underset{\mathrm{SR}\;\mathrm{SMC}\;Ca^{2+}\;\text{terms}}{\underset{︸}{J_{\mathrm{SR}\text{\_}\text{uptake}}-J_{\mathrm{CICR}}-J_{\text{leak}}}}$$

(A3) $$\begin{array}{cc} \frac{\mathrm{dv}}{\mathrm{dt}}= & \;\;\underset{\mathrm{SMC}\;\text{membrane potential terms}}{\underset{︸}{\gamma \left(-J_{\mathrm{Na}/K}-J_{\mathrm{Cl}}-2J_{\mathrm{VOCC}}-J_{\mathrm{Na}/\mathrm{Ca}}-J_{K}\right)}}\\ +\underset{\text{Coupling membrane potential terms}}{\underset{︸}{V^{\mathrm{SMC}↔\mathrm{SMC}}+V^{\mathrm{SMC}↔\mathrm{EC}}}} \end{array}$$

(A4) $$\frac{dI}{\mathrm{dt}}=\underset{{\text{Cytosolic SMC IP}}_{3}\;\text{term}}{\underset{︸}{-J_{\text{degrade}}}}+\;\;\;\;\;\underset{\text{Coupling IP}3\;\text{terms}}{\underset{︸}{J_{IP_{3}}^{\mathrm{SMC}↔\mathrm{SMC}}+J_{IP_{3}}^{\mathrm{SMC}↔\mathrm{EC}}}}$$

(A5) $$\frac{\mathrm{d\omega}}{\mathrm{dt}}=\underset{K\;\text{channel term}}{\underset{︸}{\lambda \left(K_{\text{activation}}-\omega \right)}}$$

The following equations ((A6)—(A9)) specify the dynamics for cytosolic calcium concentration ( $\tilde{c}$), SR calcium concentration ( $\tilde{s}$), membrane potential ( $\tilde{v}$) and cytosolic IP^3^ concentration ( $\tilde{I}$) for the ECs layer in a similar manner to those for the SMC.

(A6) $$\begin{array}{cc} \frac{d\tilde{c}}{\mathrm{dt}}= & \;\;\underset{{\text{Cytosolic EC Ca}}^{2+}\;\text{terms}}{\underset{︸}{{\tilde{J}}_{IP_{3}}-{\tilde{J}}_{\mathrm{ER}\text{\_}\text{uptake}}+{\tilde{J}}_{\mathrm{CICR}}-{\tilde{J}}_{\mathrm{eff}}+{\tilde{J}}_{\text{leak}}-{\tilde{J}}_{\text{cation}}+{\tilde{J}}_{0}}}\\ +\underset{\mathrm{Coupling}\;Ca^{2+}\;\mathrm{terms}}{\underset{︸}{{\tilde{J}}_{\mathrm{Ca}}^{\mathrm{EC}↔\mathrm{EC}}+{\tilde{J}}_{\mathrm{Ca}}^{\mathrm{SMC}↔\mathrm{EC}}}} \end{array}$$

(A7) $$\frac{d\tilde{s}}{\mathrm{dt}}=\underset{\mathrm{ER}\;\mathrm{EC}\;Ca^{2+}\;\text{terms}}{\underset{︸}{{\tilde{J}}_{\mathrm{ER}\text{\_}\text{uptake}}-{\tilde{J}}_{\mathrm{CICR}}-{\tilde{J}}_{\text{leak}}}}$$

(A8) $$\begin{array}{cc} \frac{d\tilde{v}}{\mathrm{dt}}= & \;\;\;\;\underset{\text{EC membrane potential terms}}{\underset{︸}{-\frac{1}{{\tilde{C}}_{m}}\left({\tilde{J}}_{K}+{\tilde{J}}_{\text{residual}}\right)}}\\ +\underset{\text{Coupling membrane potential terms}}{\underset{︸}{{\tilde{V}}^{\mathrm{EC}↔\mathrm{EC}}+{\tilde{V}}^{\mathrm{SMC}↔\mathrm{EC}}}} \end{array}$$

(A9) $$\frac{d\tilde{I}}{\mathrm{dt}}=\underset{{\text{Cytosolic EC IP}}_{3}\;\text{term}}{\underset{︸}{{\tilde{J}}_{\mathrm{PLC}}-{\tilde{J}}_{\text{degrade}}}}+\;\;\;\underset{\text{Coupling IP}3\;\text{terms}}{\underset{︸}{J_{IP_{3}}^{\mathrm{EC}↔\mathrm{EC}}+J_{IP_{3}}^{\mathrm{SMC}↔\mathrm{EC}}}}$$

The definitions of the intracellular fluxes for SMCs and ECs are provided in Appendix A.2 and Appendix A.3, respectively, while the definitions for the homocellular and heterocellular coupling terms are provided in Appendix A.4 and Appendix A.5. The complete list of algebraic constants in the SMCs and ECs equations can be found in the work of 18.

The terms specific to a single SMC or EC (intracellular dynamics) are detailed in the work of 18, while the coupling terms (intercellular dynamics) originate from the work of 27. The complete list of algebraic constants in the SMCs and ECs equations can be found in the work of 18.

### A.2 Intracellular SMC fluxes

IP^3^‐induced Ca^2+^ release:

(A10) $$J_{{\text{IP}}_{3}}=F\frac{I^{2}}{K_{r}^{2}+I^{2}}.$$

Ca^2+^‐induced Ca^2+^ release from SR into cytosol:

(A11) $$J_{\text{CICR}}=C\frac{s^{2}}{s_{c}^{2}+s^{2}}\frac{c^{4}}{c_{c}^{4}+c^{4}}.$$

Ca^2+^ transfer back into the SR via the Ca^2+^‐activated SERCA pump:

(A12) $$J_{\text{SR\_uptake}}=B\frac{c^{2}}{c_{b}^{2}+c^{2}}.$$

Ca^2+^ leak from SR:

(A13) $$J_{\text{leak}}=\mathrm{Ls}.$$

Influx of extracellular Ca^2+^ via (VOCC):

(A14) $$J_{\text{VOCC}}=G_{\text{Ca}}\frac{v-v_{\text{Ca}\;{}_{1}}}{1+e^{-\left[\left(v-v_{\text{Ca}\;{}_{2}}\right)/R_{\text{Ca}}\right]}}.$$

Efflux of Ca^2+^ via the Na^+^/Ca^2+^ exchanger:

(A15) $$J_{\text{Na/Ca}}=G_{\text{Na/Ca}}\frac{c}{c+c_{\text{Na/Ca}}}\left(v-v_{\text{Na/Ca}}\right).$$

Removal of intracellular Ca^2+^ through the plasma membrane Ca^2+^‐ATPase (PMCA) pump:

(A16) $$J_{\text{eff}}=\mathrm{Dc}\left(1+\frac{v-v_{d}}{R_{d}}\right).$$

Function of the Ca^2+^‐activated K^+^ channels determined by the cytosolic Ca^2+^ concentration:

(A17) $$K_{\text{activation}}=\frac{{\left(c+c_{\omega }\right)}^{2}}{{\left(c+c_{\omega }\right)}^{2}+\beta e^{-\left[\left(v-v_{Ca_{3}}\right)/R_{K}\right]}}.$$

Potassium efflux through the plasma membrane‐bound *B* *K*_Ca_ channels, where *ω* is the open channel probability of the Ca^2+^‐activated channels expressed as a function of *K*_*a**c**t**i**v**a**t**i**o**n*_:

(A18) $$J_{K}=G_{K}\omega \left(v-v_{k}\right).$$

Influx of chloride ions caused by the plasma membrane depolarisation:

(A19) $$J_{\mathrm{Cl}}=G_{\text{Cl}}\left(v-v_{\mathrm{Cl}}\right).$$

Constant rate efflux of potassium through the Na^+^/K^+^ pump:

(A20) $$J_{\text{Na/K}}=F_{\mathrm{Na}/K}.$$

Linear IP^3^ concentration degradation:

(A21) $$J_{\text{degrade}}=kI.$$

### A.3 Intracellular EC fluxes

IP^3^‐induced Ca^2+^ release:

(A22) $${\tilde{J}}_{{\text{IP}}_{3}}=\tilde{F}\frac{{\tilde{I}}^{2}}{{\tilde{K}}_{r}^{2}+{\tilde{I}}^{2}}.$$

Ca^2+^‐induced Ca^2+^ release from ER into cytosol:

(A23) $${\tilde{J}}_{\text{CICR}}=\tilde{C}\frac{{\tilde{s}}^{2}}{{\tilde{s}}_{r}^{2}+{\tilde{s}}^{2}}\frac{{\tilde{c}}^{4}}{{\tilde{c}}_{c}^{4}+{\tilde{c}}^{4}}.$$

Ca^2+^ transfer back into the ER via the Ca^2+^‐activated SERCA pump:

(A24) $${\tilde{J}}_{\text{ER\_uptake}}=\tilde{B}\frac{{\tilde{c}}^{2}}{{\tilde{c}}_{b}^{2}+{\tilde{c}}^{2}}.$$

Ca^2+^ leak from ER:

(A25) $${\tilde{J}}_{\text{leak}}=\tilde{L}\tilde{s}.$$

Removal of intracellular Ca^2+^ through the PMCA pump:

(A26) $${\tilde{J}}_{\text{eff}}=\tilde{D}\tilde{c}.$$

Calcium influx through non‐selective cation channels:

(A27) $${\tilde{J}}_{\text{cation}}={\tilde{G}}_{\text{cat}}\left(E_{\text{Ca}}-\tilde{v}\right)\times \frac{1}{2}\left(1+\tanh\left(\frac{\underset{10}{\log}\tilde{c}-{\tilde{m}}_{3_{\text{cat}}}}{{\tilde{m}}_{4_{\text{cat}}}}\right)\right).$$

Constituent currents determined by the potassium efflux through large and small Ca^2+^‐activated channels:

(A28) $${\tilde{J}}_{K}={\tilde{G}}_{\text{tot}}\left(\tilde{v}-{\tilde{v}}_{K}\right)\left({\tilde{J}}_{{\text{BK}}_{\text{Ca}}}-{\tilde{J}}_{{\text{SK}}_{\text{Ca}}}\right),$$

where

(A29) $${\tilde{J}}_{{\text{BK}}_{\text{Ca}}}=\frac{0.4}{2}\left(1+\tanh\left(\frac{\left(\underset{10}{\log}\tilde{c}-{\tilde{c}}_{K}\right)\left(\tilde{v}-{\tilde{b}}_{K}\right)-{ã}_{K1}}{{\tilde{m}}_{3b}{\left(\tilde{v}+{ã}_{K2}\left(\underset{10}{\log}\tilde{c}-{\tilde{c}}_{K}\right)-{\tilde{b}}_{K}\right)}^{2}+{\tilde{m}}_{\text{4b}}}\right)\right)$$

and

(A30) $${\tilde{J}}_{{\text{SK}}_{\text{Ca}}}=\frac{0.6}{2}\left(1+\tanh\left(\frac{\underset{10}{\log}\tilde{c}-{\tilde{m}}_{3s}}{{\tilde{m}}_{4s}}\right)\right).$$

Residual current comprising an inward sodium or potassium current and an outward chloride current:

(A31) $${\tilde{J}}_{\text{residual}}={\tilde{G}}_{R}\left(\tilde{v}-{\tilde{v}}_{\text{rest}}\right).$$

Linear IP^3^ concentration degradation:

(A32) $${\tilde{J}}_{\text{degrade}}=\tilde{k}\tilde{I}.$$

### A.4 Homocellular coupling

The work of 27 provides the following definitions for the homocellular coupling components of the conservation equations for the adjacent SMCs include Ca^2+^ (A33) and IP^3^ (A34) concentration fluxes as well as the membrane potential (A35).

(A33) $$J_{\text{Ca}}^{\mathrm{SMC}↔\mathrm{SMC}}=-p_{\mathrm{Ca}}\left(c-c_{k}\right)$$

(A34) $$J_{IP_{3}}^{\mathrm{SMC}↔\mathrm{SMC}}=-p_{IP_{3}}\left(I-I_{k}\right)$$

(A35) $$V^{\mathrm{SMC}↔\mathrm{SMC}}=-g\left(v-v_{k}\right)$$

Similarly, the homocellular coupling components of the conservation equations for the adjacent ECs include Ca^2+^ (A36) and IP^3^ (A37) concentration fluxes as well as the membrane potential (A38).

(A36) $$J_{\mathrm{Ca}}^{\mathrm{EC}↔\mathrm{EC}}=-{\tilde{p}}_{\mathrm{Ca}}\left(\tilde{c}-{\tilde{c}}_{l}\right)$$

(A37) $$J_{IP_{3}}^{\mathrm{EC}↔\mathrm{EC}}=-{\tilde{p}}_{IP_{3}}\left(\tilde{I}-{\tilde{I}}_{l}\right)$$

(A38) $$V^{\mathrm{EC}↔\mathrm{EC}}=-\tilde{g}\left(\tilde{v}-{\tilde{v}}_{l}\right)$$

In the above‐mentioned equations, the *k* and *l* subscripts are the indices of the adjacent SMCs and ECs, respectively.

### A.5 Heterocellular coupling

The work of 27 provides the following definitions for the heterocellular coupling components of the conservation equations for an SMC and its adjacent ECs include Ca^2+^ (A39) and IP^3^ (A40) concentration fluxes as well as the membrane potential (A41).

(A39) $$J_{\mathrm{Ca}}^{\mathrm{SMC}↔\mathrm{EC}}=-P_{\mathrm{Ca}}\left(c-c_{m}\right)$$

(A40) $$J_{IP_{3}}^{\mathrm{SMC}↔\mathrm{EC}}=-P_{IP_{3}}\left(I-I_{m}\right)$$

(A41) $$V^{\mathrm{SMC}↔\mathrm{EC}}=-G\left(v-v_{m}\right)$$

Similarly, the heterocellular coupling components of the conservation equations for an EC and its adjacent SMCs include Ca^2+^ (A42) and IP^3^ (A43) concentration fluxes as well as the membrane potential (A44).

(A42) $$J_{\mathrm{Ca}}^{\mathrm{SMC}↔\mathrm{EC}}=-{\tilde{P}}_{\mathrm{Ca}}\left(\tilde{c}-{\tilde{c}}_{n}\right)$$

(A43) $$J_{IP_{3}}^{\mathrm{SMC}↔\mathrm{EC}}=-{\tilde{P}}_{IP_{3}}\left(\tilde{I}-{\tilde{I}}_{n}\right)$$

(A44) $$V^{\mathrm{SMC}↔\mathrm{EC}}=-\tilde{G}\left(\tilde{v}-{\tilde{v}}_{n}\right)$$

In the equations above, the *m* and *n* subscripts are the indices of the ECs adjacent to a given SMC and SMCs adjacent to a given EC, respectively.

### A.6 Koenigsberger model parameters

Table A.1 provides the parameters for a single SMC, while Table A2 provides the parameters for a single EC.

**Table A.1:** SMC model parameters as described by 18.

*F*	Maximal rate of activation‐dependent calcium influx	0.23 *μ* *M*s^−1^
*K*_*r*_	Half saturation constant for agonist‐dependent calcium entry	1 *μ* *M*
*G*_*C**a*_	Whole cell conductance for VOCCs	0.00129 *μ* *M*mV^−1^s^−1^
*v*_*C**a*1_	Reversal potential for VOCCs	100 mV
*v*_*C**a*2_	Half‐point of the VOCC activation sigmoidal	−24 mV
*R*_*C**a*_	Maximum slope of the VOCC activation sigmoidal	8.5 mV
*G*_*N**a*/*C**a*_	Whole cell conductance for Na^+^/Ca^2+^ exchange	0.00316 *μ* *M*mV^−1^s^−1^
*c*_*N**a*/*C**a*_	Half‐point for activation of Na^+^/Ca^2+^ exchange by Ca^2+^	0.5 *μ* *M*
*v*_*N**a*/*C**a*_	Reversal potential for the Na^+^/Ca^2+^ exchanger	−30 mV
*B*	SR uptake rate constant	2.025 *μ* *M*s^−1^
*c*_*b*_	Half‐point of the SERCA activation sigmoidal	1.0 *μ* *M*
*C*	CICR rate constant	55 *μ* *M*s^−1^
*s*_*c*_	Half‐point of the CICR Ca^2+^ efflux sigmoidal	2.0 *μ* *M*
*c*_*c*_	Half‐point of the CICR activation sigmoidal	0.9 *μ* *M*
*D*	Rate constant for Ca^2+^ extrusion by the ATPase pump	0.24 s^−1^
*v*_*d*_	Intercept of voltage dependence of extrusion ATPase	−100 mV
*R*_*d*_	Slope of voltage dependence of extrusion ATPase	250 mV
*L*	Leak from SR rate constant	0.025 s^−1^
*g*	Scaling factor relating net movement of ion fluxes to the membrane potential (inversely related to cell capacitance)	1970 mV^−1^ *μ* *M*
*F*_*N**a*/*K*_	Net whole cell flux via the Na^+^–K^+^–ATPase	0.0432 *μ* *M*s^−1^
*G*_*C**l*_	Whole cell conductance for Cl^−^ current	0.00134 *μ* *M*mV^−1^s^−1^
*v*_*C**l*_	Reversal potential for Cl^−^ channels	−25 mV
*G*_*K*_	Whole cell conductance for K^+^ efflux	0.00446 *μ* *M*mV^−1^s^−1^
*v*_*K*_	Reversal potential for K^+^	−94 mV
*λ*	Rate constant for net K_*C**a*_ channel opening	45
*c*_*ω*_	Translation factor for Ca^2+^ dependence of K_*C**a*_ channel activation sigmoidal	0 *μ* *M*
*β*	Translation factor for membrane potential dependence of K_*C**a*_ channel activation sigmoidal	0.13 *μ* *M* ^2^
$v_{Ca_{3}}$	Half‐point for the K_*C**a*_ channel activation sigmoidal	−27 mV
*R*_*K*_	Maximum slope of the K_*C**a*_ activation sigmoidal	12.0 mV
*k*	Rate constant of IP^3^ degradation	0.1 s^−1^

**Table A2:** EC model parameters as described by 18.

$\tilde{F}$	Maximal rate of activation‐dependent calcium influx	0.23 *μ* *M*s^−1^
${\tilde{K}}_{r}$	half saturation constant for agonist‐dependent calcium entry	1 *μ* *M*
$\tilde{B}$	ER uptake rate constant	0.5 *μ* *M*s^−1^
${\tilde{c}}_{b}$	Half‐point of the SERCA activation sigmoidal	1.0 *μ* *M*
$\tilde{C}$	CICR rate constant	5 *μ* *M*s^−1^
${\tilde{s}}_{c}$	Half‐point of the CICR Ca^2+^ efflux sigmoidal	2.0 *μ* *M*
${\tilde{c}}_{c}$	Half‐point of the CICR activation sigmoidal	0.9 *μ* *M*
$\tilde{D}$	Rate constant for Ca^2+^ extrusion by the ATPase pump	0.24 s^−1^
$\tilde{L}$	Leak from SR rate constant	0.025 s^−1^
$\tilde{k}$	Rate constant of IP^3^ degradation	0.1 s^−1^
${\tilde{G}}_{\mathrm{cat}}$	Whole cell cation channel conductivity	0.66 *μ* *M* *μ* *V* ^−1^s^−1^
${\tilde{E}}_{\mathrm{Ca}}$	Ca^2+^ equilibrium potential	50 *μ* *V*
${\tilde{m}}_{3_{\mathrm{cat}}}$		−0.18 *μ* *M*
${\tilde{m}}_{4_{\mathrm{cat}}}$		0.37 *μ* *M*
${\tilde{J}}_{0}$	Constant calcium influx	0.029 *μ* *M*s^−1^
${\tilde{C}}_{m}$	Membrane capacitance	25.8 pF
${\tilde{G}}_{\mathrm{tot}}$	Total potassium channel conductivity	6927 pS
${\tilde{v}}_{K}$	K^+^ equilibrium potential	−80 *μ* *V*
${ã}_{K1}$		53.3 *μ* *M* *u* *V*
${ã}_{K2}$		53.3 *μ* *V* *μ* *M* ^−1^
${\tilde{b}}_{K}$		−80.8 *μ* *V*
${\tilde{c}}_{K}$		−0.4 *μ* *M*
${\tilde{m}}_{3b}$		1.32 ×10^3^ *μ* *M* *μ* *V* ^−1^
${\tilde{m}}_{4b}$		0.30 *μ* *M* *μ* *V* ^−1^
${\tilde{m}}_{3s}$		−0.28 *μ* *M*
${\tilde{m}}_{4s}$		0.389 *μ* *M*
${\tilde{G}}_{R}$	Residual current conductivity	955 pS
${\tilde{v}}_{\mathrm{rest}}$	Membrane resting potential	−31.1 *μ* *V*

### A.7 Coupling parameters

Table A.3 shows the coupling parameters for the four coupling configurations reported in this research.

**Table A.3:** Coupling parameters for four intercellular communication configurations.

		Case 1	Case 2	Case 3	Case 4
Homocellular	*g*	1000	1000	1000	1000
	$\tilde{g}$	1000	1000	1000	0
	*p*_*C**a*_	0.05	0.05	0.05	0.05
	${\tilde{p}}_{\mathrm{Ca}}$	0.05	0.05	0.05	0
	$p_{IP_{3}}$	0.05	0.05	0.05	0.05
	${\tilde{p}}_{IP_{3}}$	0	0	0.05	0.05
Heterocellular	*G*	50.0	50.0	50.0	0
	$\tilde{G}$	50.0	50.0	50.0	0
	*P*_*C**a*_	0	0.05	0.05	0
	${\tilde{P}}_{\mathrm{Ca}}$	0	0.05	0.05	0
	$P_{IP_{3}}$	0.05	0.05	0.05	0.05
	${\tilde{P}}_{IP_{3}}$	0.05	0.05	0.05	0.05

Cases 1 and 2 simulate healthy paracrine communication, while Cases 3 and 4 simulate pathological cases, with early and progressive atherosclerotic lesions, respectively.

## References

[1] Rohrig UF , Awad L , Grosdidier A , Larrieu P , Stroobant V , Colau D , Cerundolo V , Simpson AJG , Vogel P , Van den Eynde BJ , Zoete V , Michielin O . Rational design of indoleamine 2,3‐dioxygenase inhibitors. Journal of Medicinal Chemistry 2010; 53(3): 1172–1189. 20055453 10.1021/jm9014718

[2] Li S , Brazhnik P , Sobral B , Tyson JJ . Temporal controls of the asymmetric cell division cycle in caulobacter crescentus. PLoS Computational Biology 2009; 5(8): 1–15, doi:10.1371/journal.pcbi.1000463. 10.1371/journal.pcbi.1000463 PMC2714070 19680425

[3] Rackauskas M , Verselis VK , Bukauskas FF . Permeability of homotypic and heterotypic gap junction channels formed of cardiac connexins mCx30.2, Cx40, Cx43, and Cx45. American Journal of Physiology. Heart and Circulatory Physiology September 2007; 293(3): H1729–H1736. 17557922 10.1152/ajpheart.00234.2007 PMC2836796

[4] Bedner P , Niessen H , Odermatt B , Kretz M , Willecke K , Harz H . Selective permeability of different connexin channels to the second messenger cyclic AMP. Journal of Biological Chemistry 2006; 281(10): 6673–6681. 16373337 10.1074/jbc.M511235200

[5] Brisset AC , Isakson BE , Kwak BR . Connexins in vascular physiology and pathology. Antioxidants & Redox Signaling 2009; 11(2): 267–282. 18834327 10.1089/ars.2008.2115 PMC2819334

[6] Tran CHT , Vigmond EJ , Plane F , Welsh DG . Mechanistic basis of differential conduction in skeletal muscle arteries. The Journal of Physiology 2009; 587: 1301–1318. 19171655 10.1113/jphysiol.2008.166017 PMC2674999

[7] Kapela A , Bezerianos A , Tsoukias NM . A mathematical model of vasoreactivity in rat mesenteric arterioles: I. Myoendothelial communication. Microcirculation 2009; 16: 694–713. 19905969 10.3109/10739680903177539 PMC3547604

[8] Boileau E , Bevan RLT , Sazonov I , Rees MI , Nithiarasu P . Flow‐induced ATP release in patient‐specific arterial geometries a comparative study of computational models. International Journal for Numerical Methods in Biomedical Engineering 2013; 29(10): 1038–1056. 23894050 10.1002/cnm.2581

[9] Boileau E , Parthimos D , Nithiarasu P . 2015 An extended computational framework to study arterial vasomotion and its links to vascular disease In Biomedical Technology, Lenarz T, Wriggers P (eds)., Lecture Notes in Applied and Computational Mechanics, vol. 74 Springer International Publishing: Cham; 129–144.

[10] Cilla M , Peña E , Martínez M . Mathematical modelling of atheroma plaque formation and development in coronary arteries. Journal of the Royal Society, Interface 2014; 11(90): 20130860 –866. 10.1098/rsif.2013.0866 PMC3836327 24196695

[11] Caro CG , Fitz‐Gerald JM , Schroter RC . Atheroma and arterial wall shear observation, correlation and proposal of a shear dependent mass transfer mechanism for atherogenesis. Proceedings of the Royal Society of London B: Biological Sciences 1971; 177(1046): 109–133. 4396262 10.1098/rspb.1971.0019

[12] Malek AM , Alper SL , Izumo S . Hemodynamic shear stress and its role in atherosclerosis. The Journal of American Medical Asociation 1999; 282(21): 2035–2042. 10.1001/jama.282.21.2035 10591386

[13] Himburg HA , Grzybowski DM , Hazel AL , LaMack JA , Li XM , Friedman MH . Spatial comparison between wall shear stress measures and porcine arterial endothelial permeability. American Journal of Physiology ‐ Heart and Circulatory Physiology April 2004; 286(5): H1916–H1922. 14715506 10.1152/ajpheart.00897.2003

[14] Comerford A , David T . Computer model of nucleotide transport in a realistic porcine aortic trifurcation. Annals of Biomedical Engineering 2008; 36(7): 1175–1187. 18415019 10.1007/s10439-008-9493-0

[15] David T . Wall shear stress modulation of ATP/ADP concentration at the endothelium. Annals of Biomedical Engineering 2003; 31(10): 1231–1237. 14649496 10.1114/1.1615574

[16] Yamamoto K , Sokabe T , Ohura N , Nakatsuka H , Kamiya A , Ando J . Endogenously released ATP mediates shear stress‐induced Ca^2+^ influx into pulmonary artery endothelial cells. American Journal of Physiology ‐ Heart and Circulatory Physiology 2003; 285(2): H793–H803. 12714321 10.1152/ajpheart.01155.2002

[17] Plank MJ , Wall DJN , David T . Atherosclerosis and calcium signalling in endothelial cells. Progress in Biophysics and Molecular Biology 2006; 91: 287–313. 16171849 10.1016/j.pbiomolbio.2005.07.005

[18] Koenigsberger M , Sauser R , Bény JL , Meister JJ . Role of the endothelium on arterial vasomotion. Biophysical Journal 2005; 88: 3845–3854. 15792979 10.1529/biophysj.104.054965 PMC1305618

[19] Seppey D , Sauser R , Koenigsberger M , Beny JL , Meister JJ . Intercellular calcium waves are associated with the propagation of vasomotion along arterial strips. Am J Physiol Heart Circ Physiol 2010; 298: H488–H496. 19966061 10.1152/ajpheart.00281.2009

[20] Koenigsberger M , Seppey D , Bény JL , Meister JJ . Mechanisms of propagation of intercellular calcium waves in arterial smooth muscle cells. Biophysical Journal 2010; 99(2): 333–43. 20643050 10.1016/j.bpj.2010.04.031 PMC2905113

[21] Chen K , Popel AS . Vascular and perivascular nitric oxide release and transport: biochemical pathways of neuronal nitric oxide synthase (nos1) and endothelial nitric oxide synthase (nos3). Free Radical Biology and Medicine 2007; 42(6): 811–822. 17320763 10.1016/j.freeradbiomed.2006.12.007 PMC1987713

[22] Davignon J , Ganz P . Role of endothelial dysfunction in atherosclerosis. Circulation 2004; 109([supplement ] III): III–27–III–32. 10.1161/01.CIR.0000131515.03336.f8 15198963

[23] Olesen SP , Clapham DE , Davies PF . Haemodynamic shear stress acgtivates a k+ current in vascular endothelial cells. Nature 1988; 331: 168–170. 2448637 10.1038/331168a0

[24] Ku DN , Giddens DP , P ZD . Pulsatile flow and atherosclerosis in the human carotid bifurcation. Positive correlation between plaque location and low oscillating shear stress. Arteriosclerosis 1985; 5: 293–302. 3994585 10.1161/01.atv.5.3.293

[25] Behringer EJ , Segal SS . Tuning electrical conduction along endothelial tubes of resistance arteries through ca^2+^ activated k^+^ channels. Circulation Research 2012; 110: 1311–1321. 22492531 10.1161/CIRCRESAHA.111.262592 PMC3467972

[26] Emerson GG , Neild TO , Segal SS . Conduction of hyperpolarization along hamster feed arteries: augmentation by acetylcholine. American Journal of Physiology and Heart Circulation Physiology 2002; 283: H102–H109. 10.1152/ajpheart.00038.2002 12063280

[27] Shaikh MA , Wall DJN , David T . Macro‐scale phenomena of arterial coupled cells: a massively parallel simulation. Journal of the Royal Society Interface May 2012; 9(70): 972–87. 10.1098/rsif.2011.0453 PMC3306636 21920960

[28] Bennett MR , Farnell L , Gibson WG . A quantitative model of purinergic junctional transmission of calcium waves in astrocyte networks. Biophysical Journal 2005; 89: 2235–2250. 16055527 10.1529/biophysj.105.062968 PMC1366726

[29] Lemon G , Gibson WG , Bennett MR . Metabotropic receptor activation, desensitization and sequestration – I: modelling calcium and inositol 1,4,5‐trisphosphate dynamics following receptor activation. Journal of Theoretical Biology 2003; 223(1): 93–111. 12782119 10.1016/s0022-5193(03)00079-1

[30] Burnier L , Fontana P , Angelillo‐Scherrer A , Kwak BR . Intercellular communication in atherosclerosis. Physiology (Bethesda, Md.) 2009; 24: 36–44. 10.1152/physiol.00036.2008 19196650

[31] Bukauskas FF , Bukauskiene A , Verselis VK . Conductance and permeability of the residual state of connexin43 gap junction channels. Journal of General Physiology 2002; 119(2): 171–186. 11815667 10.1085/jgp.119.2.171 PMC2233803

[32] Van Rijen H , van Kempen MJ , Analbers LJ , Rook MB , van Ginneken AC , Gros D , Jongsma HJ . Gap junctions in human umbilical cord endothelial cells contain multiple connexins. American Journal of Physiology‐Cell Physiology 1997; 272(1): C117–C130. 10.1152/ajpcell.1997.272.1.C117 9038818

[33] Yamamoto Y , Klemm MF , Edwards FR , Suzuki H . Intercellular electrical communication among smooth muscle and endothelial cells in guinea‐pig mesenteric arterioles. Journal of Physiology 2001; 535(1): 181–195. 11507168 10.1111/j.1469-7793.2001.00181.x PMC2278769

[34] Bloor MIG , Wilson MJ . Generic parameterization of bifurcating structures In Mathematics of Surfaces, vol. 2768, Wilson MJ, Martin RR (eds)., Lecture Notes in Computer Science, 2003; 30–39.

[35] Bloor MIG , Wilson MJ . Generating blend surfaces using partial differential equations. Computer‐Aided Design 1989; 21(3): 165–171.

[36] Schroeder W , Martin K , Lorensen B . The Visualization Toolkit: An Object‐Oriented Approach to 3D Graphics (4th edn.) Kitware Inc.: Clifton Park, NY, US, 2006.

[37] Brankin RW , Gladwell I , Malvern D . Algorithm 771: rksuite_90: fortran 90 software for ordinary differential equation initial‐value problems. ACM Transactions on Mathematical Software 1997; 23(3): 402–415.

[38] Gropp W , Hoefler T , Thakur R , Lusk E . Using Advanced MPI: Modern Features of the Message‐Passing Interface. MIT Press: Cambridge, MA, US, 2014.

[39] Ayachit U . The Paraview Guide: A Parallel Visualization Application. Kitware, Inc.: Clifton Park, NY, US, 2015.

[40] Weller HG , Tabor G . A tensorial approach to computational continuum mechanics using object‐oriented techniques. Computers in Physics 1998; 12(6): 620–631.

[41] Wilkins M , Sneyd J . Intercellular spiral waves of calcium. Journal of Theoretical Biology 1998; 191(3): 299–308. 9631570 10.1006/jtbi.1997.0585

[42] Murray JD . 2003 Multi‐species waves and practical applications In Mathematical Biology II: Spatial Models and Biomedical Applications, Interdisciplinary Applied Mathematics, vol. 18, Springer New York: New York, NY; 1–70.

