# Supplementary material for: 3D time‐varying simulations of Ca2+ dynamics in arterial coupled cells: A massively parallel implementation
Source: Int J Numer Method Biomed Eng. 2016 Jul 1;33(2):e02786. doi: 10.1002/cnm.2786 (PMC5298049; doi:10.1002/cnm.2786)
Supplement: Supplementary file 1 — Supporting info item [file CNM-33-0-s001.zip › GraphicalAbstract.docx]

3D time-dependent simulations of Ca^2+^ dynamics in
coupled arterial cells: a massively parallel
implementation

Constantine Zakkaroff, Stephen Moore, Stewart Dowding, and, Tim David

This research presents a massively parallel framework for performing simulations of coupled endothelial and smooth muscle dynamics in a bifurcating artery. The simulations reported in this work include up to 2.3 million cells modelled by more than 20 million ordinary differential equations. The results of our simulations show a radically varying range of Ca^2+^ wave propagation profiles determined by the cell coupling configurations. The simulations demonstrate that heterotypic Ca 2+ diffusion through gap junctions is the dominant mass transport mechanism.
